# Heart failure drug proscillaridin A targets MYC overexpressing leukemia through global loss of lysine acetylation

**DOI:** 10.1186/s13046-019-1242-8

**Published:** 2019-06-13

**Authors:** Elodie M. Da Costa, Gregory Armaos, Gabrielle McInnes, Annie Beaudry, Gaël Moquin-Beaudry, Virginie Bertrand-Lehouillier, Maxime Caron, Chantal Richer, Pascal St-Onge, Jeffrey R. Johnson, Nevan Krogan, Yuka Sai, Michael Downey, Moutih Rafei, Meaghan Boileau, Kolja Eppert, Ema Flores-Díaz, André Haman, Trang Hoang, Daniel Sinnett, Christian Beauséjour, Serge McGraw, Noël J.-M. Raynal

**Affiliations:** 1 Département de pharmacologie et physiologie, Université de Montréal, Montréal, (Québec) Canada; 2 Sainte-Justine University Hospital Research Center (7.17.020), 3175, Chemin de la Côte-Sainte-Catherine, Montréal, (Québec) H3T 1C5 Canada; 3 Département de biochimie et biologie moléculaire, Université de Montréal, Montréal, (Québec) Canada; 4 Department of Cellular and Molecular Pharmacology, University of California, San Francisco, USA; 5 Department of Cellular and Molecular Medicine, Ottawa Institute of Systems Biology, Ottawa, (Ontario) Canada; 6 Département de Microbiologie, Infectiologie et Immunologie, Université de Montréal, Montréal, (Québec) Canada; 7 Department of Microbiology and Immunology, McGill University, Montreal, (Québec) Canada; 8 Department of Pediatrics, McGill University, Montreal, (Québec) Canada; 9 Institute of Research in Immunology and Cancer, Université de Montréal, Montreal, (Québec) Canada; 10 Département de pédiatrie, Université de Montréal, Montréal, (Québec) Canada; 11 Département Obstétrique-Gynécologie, Université de Montréal, Montréal, (Québec) Canada

**Keywords:** Cardiac glycosides, Proscillaridin A, MYC, Leukemia, Lysine acetylation, Chromatin remodelling, Lysine acetyltransferase, Leukemia stem cells

## Abstract

**Background:**

Cardiac glycosides are approved for the treatment of heart failure as Na^+^/K^+^ pump inhibitors. Their repurposing in oncology is currently investigated in preclinical and clinical studies. However, the identification of a specific cancer type defined by a molecular signature to design targeted clinical trials with cardiac glycosides remains to be characterized. Here, we demonstrate that cardiac glycoside proscillaridin A specifically targets MYC overexpressing leukemia cells and leukemia stem cells by causing MYC degradation, epigenetic reprogramming and leukemia differentiation through loss of lysine acetylation.

**Methods:**

Proscillaridin A anticancer activity was investigated against a panel of human leukemia and solid tumor cell lines with different MYC expression levels, overexpression in vitro systems and leukemia stem cells. RNA-sequencing and differentiation studies were used to characterize transcriptional and phenotypic changes. Drug-induced epigenetic changes were studied by chromatin post-translational modification analysis, expression of chromatin regulators, chromatin immunoprecipitation, and mass-spectrometry.

**Results:**

At a clinically relevant dose, proscillaridin A rapidly altered MYC protein half-life causing MYC degradation and growth inhibition. Transcriptomic profile of leukemic cells after treatment showed a downregulation of genes involved in MYC pathways, cell replication and an upregulation of hematopoietic differentiation genes. Functional studies confirmed cell cycle inhibition and the onset of leukemia differentiation even after drug removal. Proscillaridin A induced a significant loss of lysine acetylation in histone H3 (at lysine 9, 14, 18 and 27) and in non-histone proteins such as MYC itself, MYC target proteins, and a series of histone acetylation regulators. Global loss of acetylation correlated with the rapid downregulation of histone acetyltransferases. Importantly, proscillaridin A demonstrated anticancer activity against lymphoid and myeloid stem cell populations characterized by MYC overexpression.

**Conclusion:**

Overall, these results strongly support the repurposing of proscillaridin A in MYC overexpressing leukemia.

**Electronic supplementary material:**

The online version of this article (10.1186/s13046-019-1242-8) contains supplementary material, which is available to authorized users.

## Background

MYC (c-MYC) transcription factor is a driver of oncogenic programs. It contributes to gene deregulation in cancer by promoting expression of genes involved in cell proliferation [1]. High MYC expression drives tumor initiation, progression, and maintenance and is associated with aggressive cancers and poor prognoses [2]. MYC is a potent driver in leukemia inducing cell proliferation and blocking cell differentiation [3]. Moreover, MYC contributes to long-term self-renewal of leukemic stem cells [4]. Conversely, genetic suppression of MYC in transgenic mouse models induces differentiation and cell growth arrest of leukemic cells [5, 6]. Therefore, targeting MYC addiction in leukemia is a major therapeutic goal. Since MYC lacks a catalytic site, its direct inhibition has been extremely challenging. Indirect MYC inhibition demonstrated therapeutic efficacy with bromodomain inhibitors (such as JQ1 or THZ1), by blocking MYC transcriptional effects [7–9]. Unfortunately, cancer cells, such as leukemia, breast and ovarian cancers, develop resistance to these inhibitors by compensatory mechanisms using other bromodomain containing proteins or kinome reprogramming [10, 11]. Together, these studies highlight the need to develop new strategies to abrogate MYC addiction in cancer.

MYC stability is regulated by post-translational modifications and MYC acetylation increases its stability [12]. The deposition of acetyl groups on lysine residues is catalyzed by lysine acetyltransferases (KATs), which acetylate also histone proteins causing chromatin opening and gene activation [13]. Pharmacological inhibition of KATs represents an interesting strategy to target indirectly MYC by blocking upstream mechanisms involved in its stability. However, KATs have overlapping targets and commercially available KAT inhibitors require further optimization [14].

Recently, we reported that cardiac glycosides, which are Na^+^/K^+^ pump inhibitors and approved for heart failure treatment, exhibit significant epigenetic and anticancer effects [15, 16]. Cardiac glycosides, including digitoxin, digoxin, lanatoside C, ouabain and proscillaridin A, triggered reactivation of epigenetically silenced tumor suppressor genes, supporting their repurposing potential [16]. Interestingly, all cardiac glycosides produced synergistic responses when used in combination with the epigenetic drug decitabine (demethylating agent), further supporting their epigenetic activity [15]. Several epidemiological studies argue in favor of repurposing cardiac glycosides in oncology. Indeed, patients treated with cardiac glycosides for heart failure have a lower rate of cancer diagnosis as compared to the general population and upon cancer diagnosis; these patients exhibit generally a less aggressive disease and respond better to therapy [17]. However, the repurposing of cardiac glycosides in oncology is limited by their narrow therapeutic window. Indeed, maximal plasmatic level is around 10 nanomolar, due to cardiac toxicities [17–19]. Several in vitro and in vivo studies tested their anticancer activity at supra-pharmacological doses, which are not reachable in humans; in particular, in rodents who can tolerate high doses of these drugs due to structural differences in Na^+^/K^+^ pump as compared to human [19–21]. Since the repurposing of cardiac glycosides is restricted to the low nanomolar range, we sought to identify cancer types highly sensitive to these drugs. To do so, we screened a panel of human cancer cell lines with proscillaridin A, which was identified as the most potent cardiac glycoside in our previous screens [15, 16]. Proscillaridin A produced antiproliferative effects with a preferential selectivity towards MYC overexpressing leukemia cells. We demonstrated that proscillaridin A produced a global loss of acetylation in chromatin and MYC itself, producing epigenetic effects and MYC downregulation. These results provide compelling evidence for the repurposing of cardiac glycoside proscillaridin A against leukemia driven by MYC oncogenic signature.

## Materials and methods

### Cell culture and drug treatments

A panel of 14 human cancer cell lines and *hTERT/SV40ER-*immortalized human primary fibroblasts were used in the study. Leukemia cell lines MOLT-4 and REH were transduced with MYC lentivirus and *hTERT/SV40ER-*immortalized human primary fibroblasts were transduced with *MYC, RAS*^*V12*^ or *MYC* and *RAS*^*V12*^ lentiviruses. *MYC* lentivirus MYC_pLX307 was a gift from William Hahn & Sefi Rosenbluh (Addgene plasmid # 98363). *RAS*^*V12*^ lentivirus was a gift from Dr. Christian Beauséjour laboratory. Cell types and culture conditions are described in Additional file 1 Supplementary Materials and Methods. Proscillaridin A was purchased from Santa Cruz Biotechnologies (CAS number: 466–06-8; purity ≥90%) and cycloheximide was purchased from Acros Organics (CAS: 66–81-9; purity ≥95%). IC_50_ values were calculated with GraphPad Prism software.

### Protein and histone extractions

Whole cell proteins were extracted using cold whole-cell lysis buffer (50 mM Tris-Cl pH 7.4, 5 mM EDTA, 250 mM NaCl, 50 mM NaF, 0.1% Triton, 0.1 mM Na_3_VO_4_, and 1 mM PMSF), supplemented with Complete™ Protease Inhibitor Cocktail (Roche). Histones were harvested using acid-extraction method with cold Triton Extraction Buffer (TEB; 0.5% Triton, 2 mM PMSF, 0.02% NaN_3_, 10 mM sodium butyrate), supplemented with protease inhibitor cocktail. Protein extracts were separated by SDS-PAGE and transferred onto a polyvinyl difluoride membrane. All experiments were performed in triplicate. Densitometric analysis was performed using ImageJ software. ANOVA tests were performed with GraphPad Prism software. Antibodies are listed in the Additional file 1 Supplementary Materials and Methods section.

### RNA extraction, sequencing and analysis

QIAshredder was used to homogenize cell lysates and eliminate debris prior to RNA extraction using RNeasy Mini Kit. Briefly, 10 μg of purified RNA was treated with DNAse and quantified by Agilent RNA 6000 Nano kit bioanalyser chips. 1 μg of mRNA was used for library preparation with TruSEq Stranded mRNA LT. RNA sequencing was performed using HiSeq 2500. Experiments were performed in triplicate. Reads were aligned to human genome (hg19) using STAR v2.4.2 and differential gene expression analysis between untreated and treated cells was done using DESeq2 v1.10.1 [22, 23]. For bioinformatics analyses, data were processed using gene set enrichment analyses (GSEA, broadinstitute.org/gsea), metascape (metascape.org) and gene mania (genemania.org). MOLT-4 cells H3K27ac ChIP-seq results from publicly available dataset (GEO: GSM2037790) were associated with transcriptomic data. GSEA analysis of 8227 AML fractions and the leukemia stem cell (LSC) signatures were performed using the control sample data from GSE55814. GEO2R was used to generate a ranked list of LSC-related genes (6 LSC CD34^+^CD38^−^ samples vs 12 non-LSC CD34^−^ samples) used in GSEA analysis.

### Acetylation analysis by immunoprecipitation and mass spectrometry

Whole cell protein extracts were incubated overnight with 5 μg/ml of MYC antibody (Abcam, AB32072). After immunoprecipitation and transfer, proteins were probed with lysine pan-acetyl antibody (1:2500 Cell Signaling 9681). For acetylome analysis by mass spectrometry, samples were prepared as previously described [24]. Briefly, 4 biological replicates of untreated and proscillaridin A-treated MOLT-4 cells (5 nM, 48 h) were digested with trypsin. Peptides were analyzed by mass-spectrometry and data were extracted with the MaxQuant software package (version 1.5.5.1) and subsequently analyzed using an in-house computational pipeline for statistical analysis of relative quantification with fixed and/or mixed effect models, implemented in the MSstats Bioconductor package (version 3.3.10) [25, 26]. Peptides were searched with SwissProt human protein database.

## Results

### Cardiac glycoside Proscillaridin A targets MYC-driven leukemic cells

**Fig. 1:**
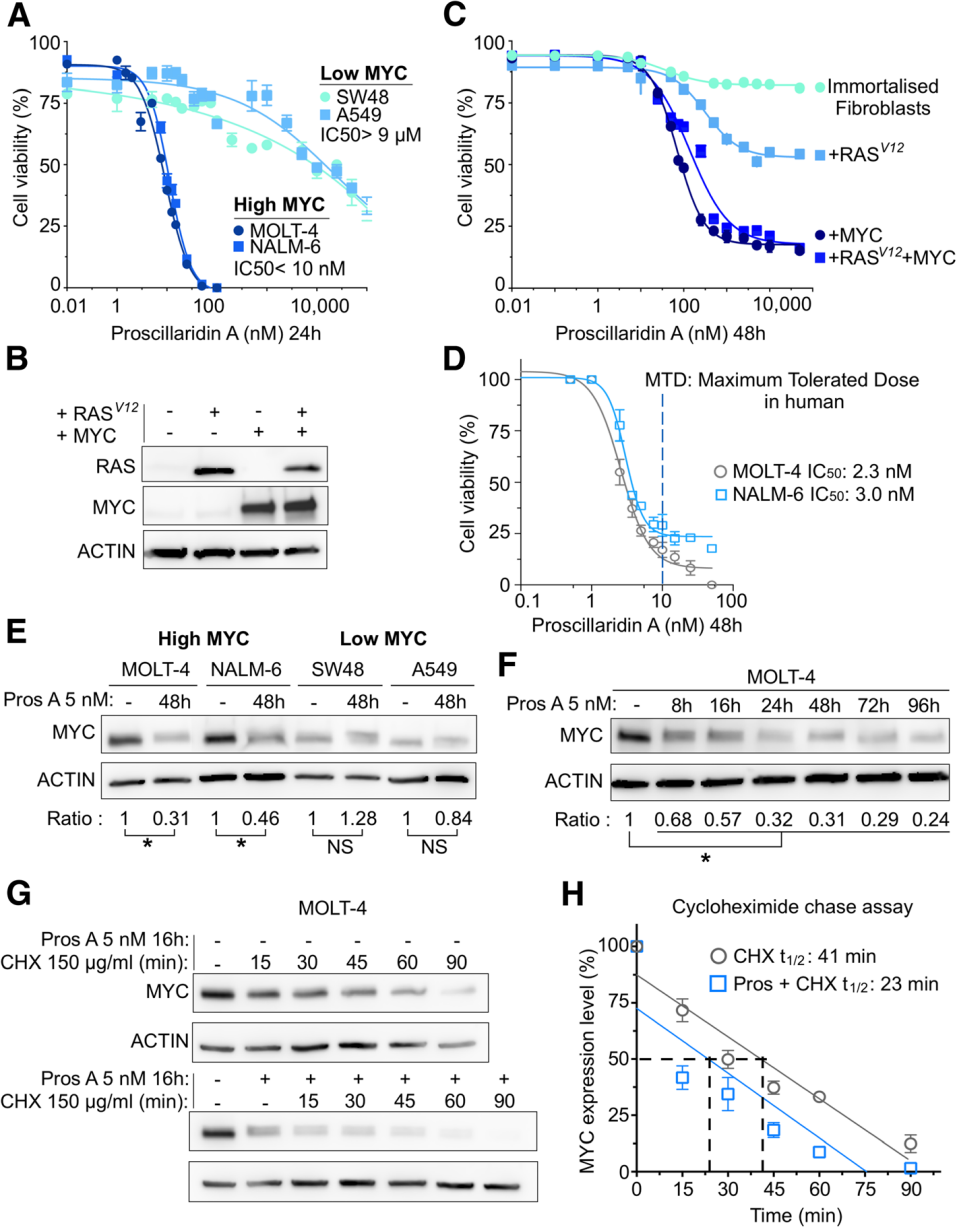
Targeting High MYC Expressing Cancer Cells with Cardiac Glycoside Proscillaridin A. **a** Cell viability and half-maximal inhibitory concentration (IC_50_) calculations after a 24 h proscillaridin A treatment (ranging from 1 nM to 100 μM) in a high MYC expressing human leukemia cell lines (MOLT-4 and NALM-6) and in low MYC expressing human tumor cell lines (SW48 and A549) (*n* = 4). **b** *MYC* and *RAS* protein expression assessed by western blotting in immortalized fibroblasts and fibroblasts transduced with *RAS*^*V12*^, *MYC* and *RAS*^*V12*^/*MYC* (*n* = 3). **c** Dose response curves after 48 h proscillaridin A treatment (0.01 nM to 50 μM) in immortalized fibroblasts and fibroblasts transduced with *RAS*^*V12*^, *MYC* and *RAS*^*V12*^/*MYC* (*n* = 4). **d** Dose response curves and IC_50_ values after 48 h proscillaridin A treatment (ranging from 0.1 nM to 100 nM) in MOLT-4 (T-cell leukemia), and NALM-6 (B-cell leukemia) (*n* = 3). Maximum tolerated dose in human is indicated. **e** MYC protein expression after proscillaridin A treatment (5 nM; 48 h) in MOLT-4, NALM-6, SW48 (colon cancer) and A549 (lung cancer) cells assessed by western blotting. MYC expression is calculated as a ratio over ACTIN levels (*indicates *P* < 0.05; One-way ANOVA; *n* = 3). **f** Time course experiment in MOLT-4 cells treated with proscillaridin A at 5 nM (8 h to 96 h). MYC protein expression is calculated as a ratio over ACTIN levels (*indicates *P* < 0.05; One-way ANOVA; *n* = 3). **g** Effect of proscillaridin A on MYC half-life in MOLT-4 cells. MYC half-life was estimated by cycloheximide (CHX) treatment (150 μg/ml) in MOLT-4 cells pretreated or not with proscillaridin A (5 nM, 16 h). MYC protein expression was performed by Western blotting and ACTIN expression was used as loading control. **h** MYC expression level after cycloheximide treatment (150 μg/ml) was quantified over ACTIN levels and expressed relative to the level at time zero (grey) or expressed relative to the level following proscillaridin A treatment (5 nM, 16 h; blue). Linear regression analysis was conducted and MYC half-life was calculated

To identify cancer types with high sensitivity to proscillaridin A in order to obtain concentrations within their therapeutic window, we screened a panel of 14 human cancer cell lines and measured cancer cell proliferation after a 24 h treatment. After calculating the half-maximal inhibitory concentration (IC_50_), we noticed a 2800-fold difference in IC_50_ values_,_ with leukemic cells being more sensitive to proscillaridin A (Additional file 2: Figure S1A). To explore the cause of this striking difference, we hypothesized that the oncogenic context in leukemia cells might influence drug efficacy. Since MYC is often overexpressed in leukemia, we compared MYC protein expression (in untreated cancer cells) with proscillaridin A IC_50_ values. Interestingly, we found a significant inverse correlation between MYC expression and proscillaridin A IC_50_ values (*p* = 0.0172; Additional file 2: Figure S1A and S1B). Proscillaridin A produced a more potent growth inhibition in cells expressing high levels of MYC protein, such as acute lymphoblastic T-cell (MOLT-4) and B-cell (NALM-6) leukemia while being less effective in colorectal (SW48) and lung (A549) cancer cells expressing low levels of MYC (Fig. 1a).

To evaluate MYC contribution to proscillaridin A therapeutic efficacy in cancer cells, we investigated drug response using an isogenic cell system consisting of *hTERT/SV40ER-*immortalized human primary fibroblasts, transformed with different oncogenes including *MYC, RAS*^*V12*^ or the combination of both oncogenes. This system allowed exploring the effect of oncogenic transformation within the same genetic background. After transduction, *MYC*-transformed fibroblasts had a small and round phenotype whereas *RAS*^*V12*^*-*transformed cells displayed increased vacuole formation and large cytoplasm. *MYC* and *RAS*^*V12*^-transformed cells exhibited a round phenotype with vacuole formation in the cytoplasm (Additional file 2: Figure S1C). High levels of MYC and RAS protein were detected after transductions when compared to non-transduced cells (Fig. 1b). Using a wide range of proscillaridin A concentrations (from 0.01 nM to 100 μM) for 48 h, we measured cell viability and calculated IC_50_ values (Fig. 1c). Untransformed fibroblasts were fully resistant to proscillaridin A. Likewise, *RAS*^*V12*^-transformed cells were mildly affected by the treatment where proscillaridin A at high doses failed to impact cell viability by more than 50%. Conversely, *MYC* transformed fibroblasts were highly sensitive to proscillaridin A with an IC_50_ value of 70 nM. Moreover, *MYC* and *RAS*^*V12*^ transformed fibroblasts (referred as to *RAS*^*V12*^ *+ MYC*) had a low IC_50_ value (132 nM) despite the presence of *RAS*^*V12*^. Consequently, we demonstrated that *MYC* overexpression is causing proscillaridin A sensitivity in transformed fibroblasts. In comparison, after 48 h treatment, MOLT-4 and NALM-6 cells (MYC overexpressing leukemic cells) showed IC_50_ values of 2.3 nM and 3 nM, respectively (Fig. 1d). Interestingly, both after 24 h (Fig. 1a) and 48 h (Fig. 1d) treatment, proscillaridin A produced IC_50_ values below 10 nM, which correspond to clinically achievable concentrations measured in the plasma of heart failure patients. To further confirm the role of MYC protein levels to proscillaridin A sensitivity, we transduced high MYC expressing (MOTL-4) and low MYC expressing (REH) leukemia cells, with MYC oncogene. After transduction and selection steps, MOLT-4 cells had similar MYC protein levels than WT cells, most likely due to an increased cell death upon additional MYC expression (Additional file 2: Figure S1D). In these cells, proscillaridin A sensitivity was unchanged (Additional file 2: Figure S1D). However, *MYC-*transduced REH cells expressed higher MYC levels, which increased proscillaridin A sensitivity (Additional file 2: Figure S1E). Altogether, these results support proscillaridin A repurposing against MYC overexpressing leukemia.

To explore the mechanism by which *MYC* overexpression in cancer cells correlates with proscillaridin A sensitivity, we compared its effects between *MYC* driven leukemic cells (MOLT-4 and NALM-6) and low expressing *MYC* cancers driven by *KRAS* mutations (SW48 colon and A549 lung cancer cells). We found that proscillaridin A (5 nM; 48 h) significantly reduced MYC protein level by more than 50% in MOLT-4 and NALM-6 cells but not in SW48 and A549 cells (Fig. 1e). Time-course experiments with both leukemic cell lines (8 h to 96 h) showed that proscillaridin A induced a significant (up to 80%) and rapid MYC downregulation (Fig. 1f; Additional file 2: Figure S1F). These results demonstrate that low dose proscillaridin A inhibits efficiently leukemia growth causing rapid MYC downregulation. Lastly, we performed a cycloheximide-chase assay in MOLT-4 cells treated with proscillaridin A at low dose (5 nM; 16 h). We observed that drug treatment produced almost a 50% reduction in MYC protein half-life (MYC t_1/2_: 41 min in untreated cells vs 23 min in proscillaridin A treated cells; Fig. 1g and h). Therefore, proscillaridin A targets MYC overexpressing leukemia cells by reducing MYC protein half-life, causing its rapid degradation.

### Proscillaridin A downregulates cell proliferation programs and induces T-cell differentiation

**Fig. 2:**
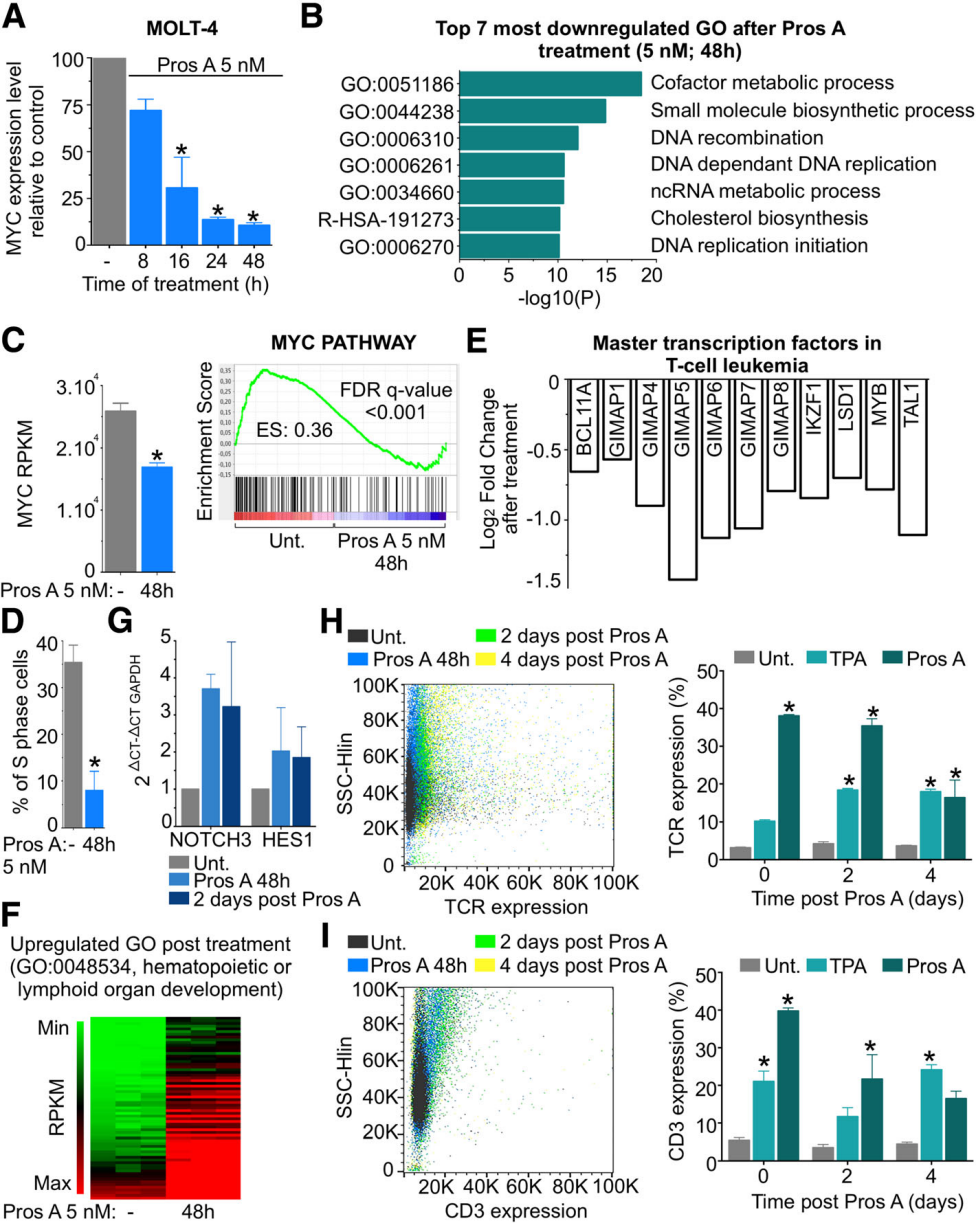
Transcriptomic Profiles from Replicative To Differentiated Phenotype After Low Dose Proscillaridin A Treatment in High MYC Expressing Leukemic Cells. **a** Quantitative PCR (qPCR) analysis of *MYC* mRNA expression after proscillaridin A treatment (5 nM; 8 h to 48 h) in MOLT-4 cells, relative to untreated cells and normalized to β-2-microglobulin (*indicates *P* < 0.05; One-way ANOVA; *n* = 3). **b** Transcriptomic analysis by RNA-Sequencing of untreated and proscillaridin A-treated (5 nM; 48 h) MOLT-4 cells (*n* = 3). Genes downregulated by proscillaridin A treatment (Log_2_ FC < -0.5) were analyzed by Metascape and the top 7 Gene Ontology (GO) pathways are displayed. **c** Left panel, MYC transcript (RPKM) expression after proscillaridin A treatment (5 nM; 48 h) in RNA-sequencing data set (*indicates *P* < 0.02, paired t-test, *n* = 3). Right panel, gene set enrichment analysis of MYC pathway before and after proscillaridin A treatment (5 nM; 48 h) in MOLT-4 cells. Enrichment score (ES) and false discovery rate (FDR) rates are shown on the graph. **d** Effect of proscillaridin A treatment (5 nM; 48 h) on the percentage of S phase cell population on MOLT-4 cells (* indicates *P* < 0.017, paired t-test, *n* = 3). **e** Gene expression values (Log_2_ fold change) obtained from RNA-sequencing in 11 genes downregulated after proscillaridin A treatment (5 nM; 48 h) in MOLT-4 cells. These genes were selected due to their role as master transcription factors associated in T-cell leukemia. **f** Heat map of gene expression levels (RPKM) involved in differentiation pathways (MOLT-4 cells) before and after proscillaridin A treatment (5 nM; 48 h). **g** Quantitative PCR (qPCR) analysis of *NOTCH3* and *HES1* mRNA expression measured after proscillaridin A treatment (5 nM; 48 h) and 2 days post treatment, relative to untreated cells and normalized to GAPDH in MOLT-4 cells (*n* = 2). **h** and **i** Left panel, T-cell differentiation markers TCR and CD3 are measured by flow cytometry in MOLT-4 cells after proscillaridin A treatment (5 nM; 48 h), as well as 2 and 4 days post treatment (*n* = 3). Right panel, percentage of TCR and CD3 expression in MOLT-4 cells. TPA treatment (10 nM; 48 h, followed by a 24 h resting period) was used as positive control of T-cell differentiation (*indicates *P* < 0.05; Two-way ANOVA; *n* = 3)

To gain insight into proscillaridin A effects against MYC-driven leukemic cells, we investigated drug-induced gene expression changes in T-ALL cells (MOLT-4). By quantitative RT-PCR (qPCR), we found that proscillaridin A significantly downregulated MYC mRNA after 16 h treatment and up to 90% after 48 h (Fig. 2a). Then, we used RNA-sequencing to explore transcriptomic effects of proscillaridin A (5 nM; 48 h) in MOLT-4 cells. After drug treatment, transcriptome analysis showed a downregulation of 2759 genes (log_2_FC < 0.5; *P*-value adjusted < 0.05) and concomitant upregulation of 3271 genes (log_2_FC > 1; *P*-value adjusted < 0.05; Additional file 3: Figure S2A and S2B). Using Metascape, gene ontology analysis revealed that downregulated genes were involved in DNA replication, biosynthesis and metabolic processes (Fig. 2b; Additional file 3: Figure S2C). Consistent with qPCR results, *MYC* transcript was significantly downregulated in our RNA-sequencing data set (Fig. 2c). Gene Set Enrichment Analysis showed that MYC PATHWAY (which includes 30 MYC target genes) was significantly downregulated (Fig. 2c). Notably, these transcriptomic effects correlated with a 25% decrease of S-phase cells as measured by BrdU staining (Fig. 2d; Additional file 3: Figure S2D). Proscillaridin A also significantly downregulated 11 T-cell leukemia master transcription factors (Fig. 2e) [27–29]. These data support that proscillaridin A efficiently inhibits proliferation programs in MYC-driven leukemia.

Gene ontology analysis also revealed that upregulated genes were enriched for hematopoietic and lymphoid organ development, suggesting the onset of leukemia differentiation (Fig. 2f and Additional file 3: Figure S2E). To probe the functional significance of this change, we measured T-cell differentiation markers in MOLT-4 cells before and after treatment. By qPCR, mRNA levels of T-cell differentiation markers *NOTCH3* and its target *HES1* were upregulated after 48 h treatment and remained expressed for 2 days after drug removal (Fig. 2g) [30]. By flow cytometry, we measured a significant increase in TCR and CD3 expression, which lasted up to 4 days after drug removal, suggesting the onset of normal T-cell activation (Fig. 2h and i) [31]. Upregulation of these differentiation markers were in the same range than the levels measured after TPA treatment, a well-known inducer of leukemia differentiation (Fig. 2h and i) [32]. Altogether, proscillaridin A treatment produced a transcriptomic shift from a proliferative program to the induction of T-cell leukemia differentiation.

### Proscillaridin A induces global loss of histone H3 acetylation

**Fig. 3:**
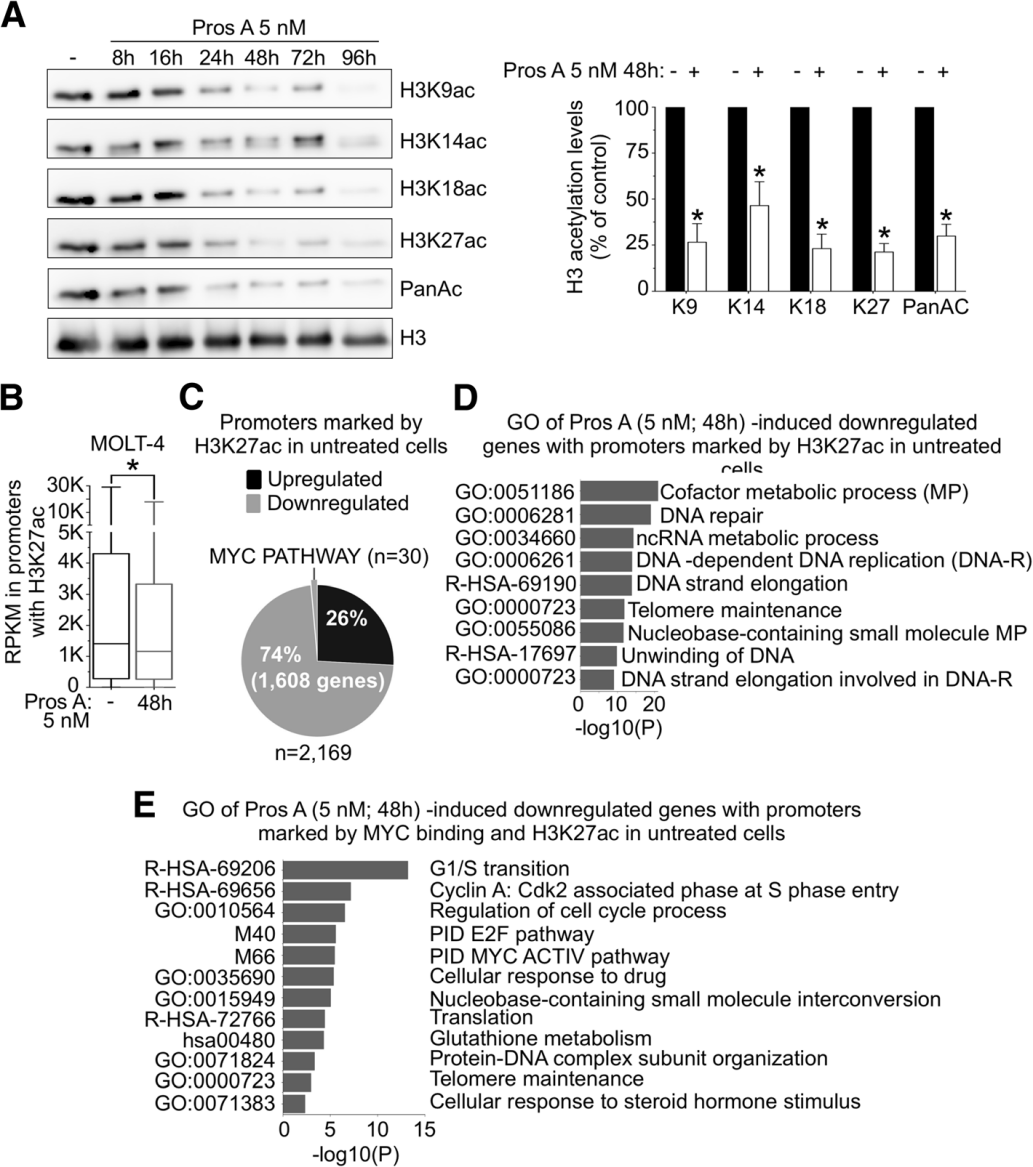
Gene Reprogramming Induced by Proscillaridin A Is Associated with Global Acetylation Loss in Histone H3. **a** Left panel, MOLT-4 cells were treated with proscillaridin A (5 nM) and histones were acid-extracted after 8, 16, 24, 48, 72 and 96 h. Histone 3 acetylation levels were assessed using antibodies against K9 ac, K14 ac, K18 ac, K27 ac, and total histone 3 acetylation. H3 was used as loading control. Right panel, H3 acetylation levels at 48 h treatment were quantified and expressed as a percentage of untreated cells (* indicates *P* < 0.05; Two-way ANOVA; *n* = 3). **b** 2169 genes are marked by H3K27ac in promoter regions (− 500/+ 500 bp) in untreated MOLT-4 cells. RPKM values of differentially expressed genes (FC > 1; FC < -0.5) from RNA-sequencing data before and after proscillaridin A treatment are displayed (* indicates *P* < 0.006; paired t-test; *n* = 3). **c** Pie chart shows percentage of upregulated (black) and downregulated (grey, including 30 MYC targets) genes after treatment (5 nM, 48 h) of genes marked by H3K27ac and MYC binding in promoters of untreated MOLT-4 cells. **d** Metascape analysis of genes marked by H3K27ac in promoters in untreated MOLT-4 cells and downregulated after proscillaridin A treatment (5 nM; 48 h). Top 9 GO pathways are displayed. **e** Metascape analysis of the 30 MYC target genes

Since proscillaridin A induced gene expression and phenotypic changes, we hypothesized that it triggers epigenetic effects in high MYC-driven leukemia. We analyzed histone H3 and H4 post-translational modifications by western blotting after 16 h to 96 h of proscillaridin A treatment (5 nM). We found that proscillaridin A produced a significant time-dependent reduction (by 75%) of lysine acetylation at H3K9, H3K14, H3K18, H3K27 residues and global loss of H3 acetylation in MOLT-4 cells (Fig. 3a; Additional file 4: Figure S3A). The dramatic reduction in H3K27ac level was confirmed by chromatin immunoprecipitation in which H3K27ac antibody pulled-down similar levels of DNA than IgG after treatment (Additional file 4: Figure S3B). Similar results were obtained in NALM-6 cells after proscillaridin A treatment (Additional file 4: Figure S3C). No change was detected on H4 acetylation or H3 methylation marks (Additional file 4: Figure S3D, S3E, Additional file 5: Figure S4A and S4B). Interestingly, loss of H3 acetylation induced global chromatin reorganization in MOLT-4 cells after treatment, as shown by DAPI staining (Additional file 5: Figure S4C).

We next asked if there was a correlation between loss of H3 acetylation and gene expression changes after proscillaridin A treatment. To address this question, we combined our RNA-Seq data pre- and post-treatment with H3K27ac ChIP-seq data of untreated MOLT-4 since this mark is associated with transcribed regions and is globally lost after treatment [17]. Among 7097 genes marked at their promoters with H3K27ac (− 500 to + 500 bp) in untreated MOLT-4 cells, 2169 genes were differentially expressed after proscillaridin A treatment. Seventy-four percent of those (1608 genes), marked by H3K27ac in untreated cells, were significantly downregulated after treatment (Fig. 3b), which is consistent with the loss of this active epigenetic mark (Fig. 3c). Gene ontology analysis of these 1608 downregulated genes showed a significant relationship with metabolism and proliferation processes (Fig. 3d; Additional file 6: Figure S5A). Among these genes, all MYC PATHWAY genes (*n* = 30) previously described (Fig. 2c) were marked by H3K27ac in untreated MOLT-4 cells and were all downregulated by treatment (Fig. 3c and e). Network analysis showed that these MYC target genes are co-expressed simultaneously, and are known to exhibit protein-protein interactions with MYC, confirming the global effect of proscillaridin A on MYC pathway (Additional file 7: Figure S6A and S6B). By contrast, upregulated genes marked by H3K27ac in untreated cells were associated with apoptosis, negative regulation of proliferation and cell differentiation (Additional file 6: Figure S5B) corroborating our functional analyses. Collectively, these results demonstrate that proscillaridin A produces global loss of H3 acetylation, which was associated with silencing of genes involved in proliferation and MYC pathway.

### Proscillaridin A induces loss of lysine acetylation in MYC target genes and chromatin regulators

**Fig. 4:**
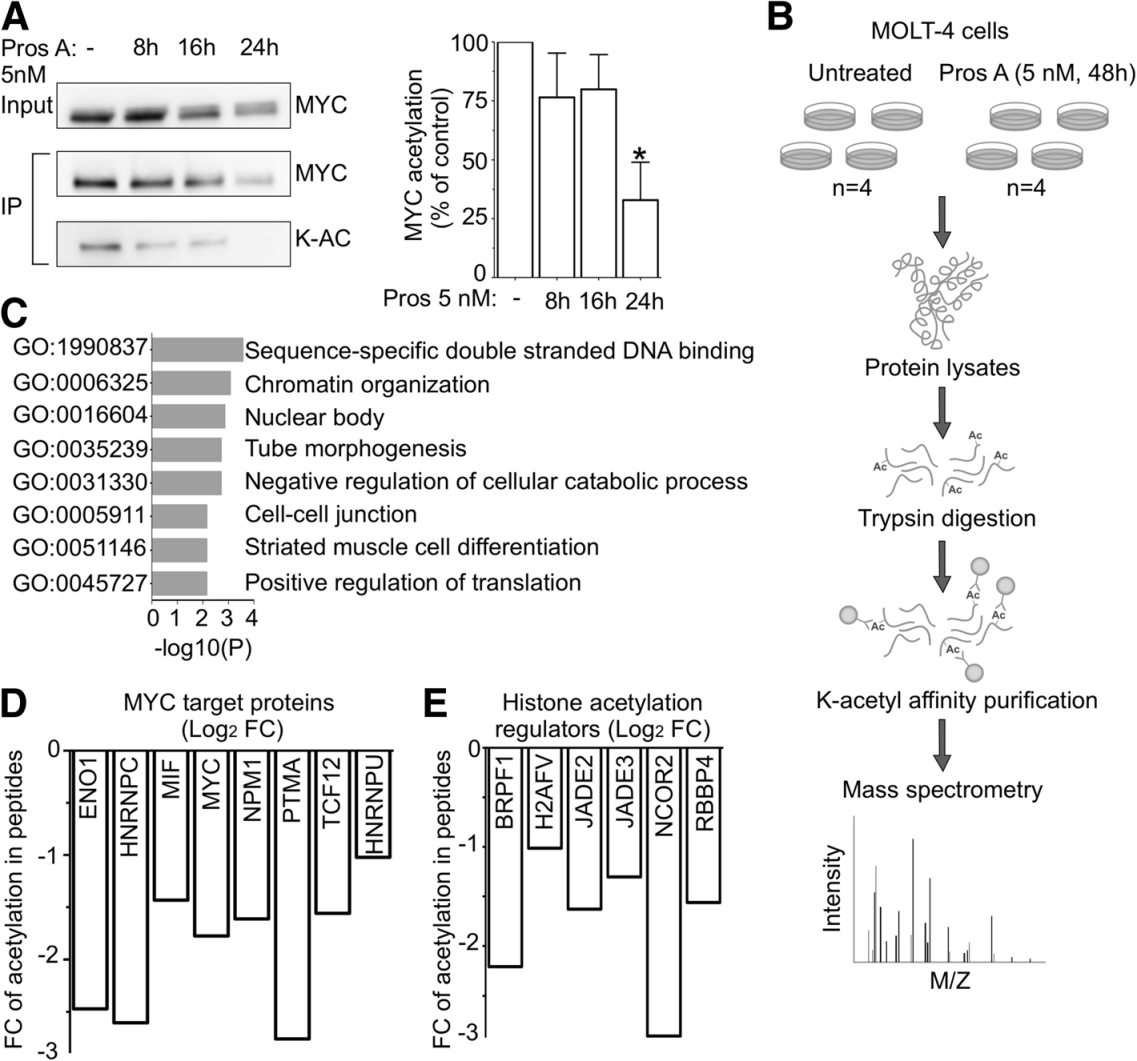
Acetylation Decrease In MYC Targets And Chromatin Regulators Induced by proscillaridin A In High MYC Expressing Cells. **a** Left panel, immunoprecipitation (IP) of MYC total lysine acetylation (K-AC) after proscillaridin A treatment (5 nM; 8 h–16 h-24 h) in MOLT-4 cells. Right panel, total lysine acetylation level on MYC was quantified and expressed as a percentage of untreated cells (* indicates *P* < 0.05; One-way ANOVA; *n* = 3). **b** Lysine acetylome profiling of MOLT-4 cells before and after proscillaridin A treatment (5 nM; 48 h). **c** Lysine acetylome metascape analysis in 28 peptides with significant loss of acetylation (Log_2_FC < − 1) after proscillaridin A treatment (5 nM; 48 h) in MOLT-4 cells. **d** Log_2_FC of acetylation levels in MYC target proteins (untreated VS treated). **e** Log_2_FC of acetylation levels of histone regulators (untreated VS treated)

We then asked whether depletion of lysine acetylation was extended to non-histone proteins after treatment. First, we measured MYC acetylation levels after 8, 16 and 24 h of proscillaridin A treatment (5 nM) in MOLT-4 cells, since this post-translational modification plays a role in its stability [12, 33]. After MYC immunoprecipitation and probing with a pan-acetyl antibody, we measured a time dependent decrease (up to 75%) of MYC total acetylation (Fig. 4a).

To further characterize the extent of acetylation loss, we conducted an acetylome study by mass spectrometry on untreated and proscillaridin A-treated (5 nM; 48 h) MOLT-4 cells (Fig. 4b). Two distinct MYC peptides showed a significant reduction in lysine acetylation after treatment, which confirmed our immunoprecipitation results (Additional file 8: Figure S7A). Mass spectrometry analysis showed that 28 peptides (including MYC) had a significant loss of lysine acetylation after treatment, associated with chromatin organization (Fig. 4c). Among them, 8 are known MYC target proteins and 6 are involved in chromatin organization (Fig. 4d and e). Networks analysis showed that these 28 proteins are generally co-expressed, suggesting a connection between their acetylation and expression levels (Additional file 8: Figure S7B). Interestingly, 8 out of 28 proteins were MYC target proteins, including MYC itself and 6 out of 28 are involved in histone acetylation regulation (Fig.4d and e). Altogether, proscillaridin A reduces lysine acetylation of MYC, its protein partners and several histone acetylation regulators.

### Proscillaridin A efficiently downregulates histone acetyltransferases involved in MYC acetylation

**Fig. 5:**
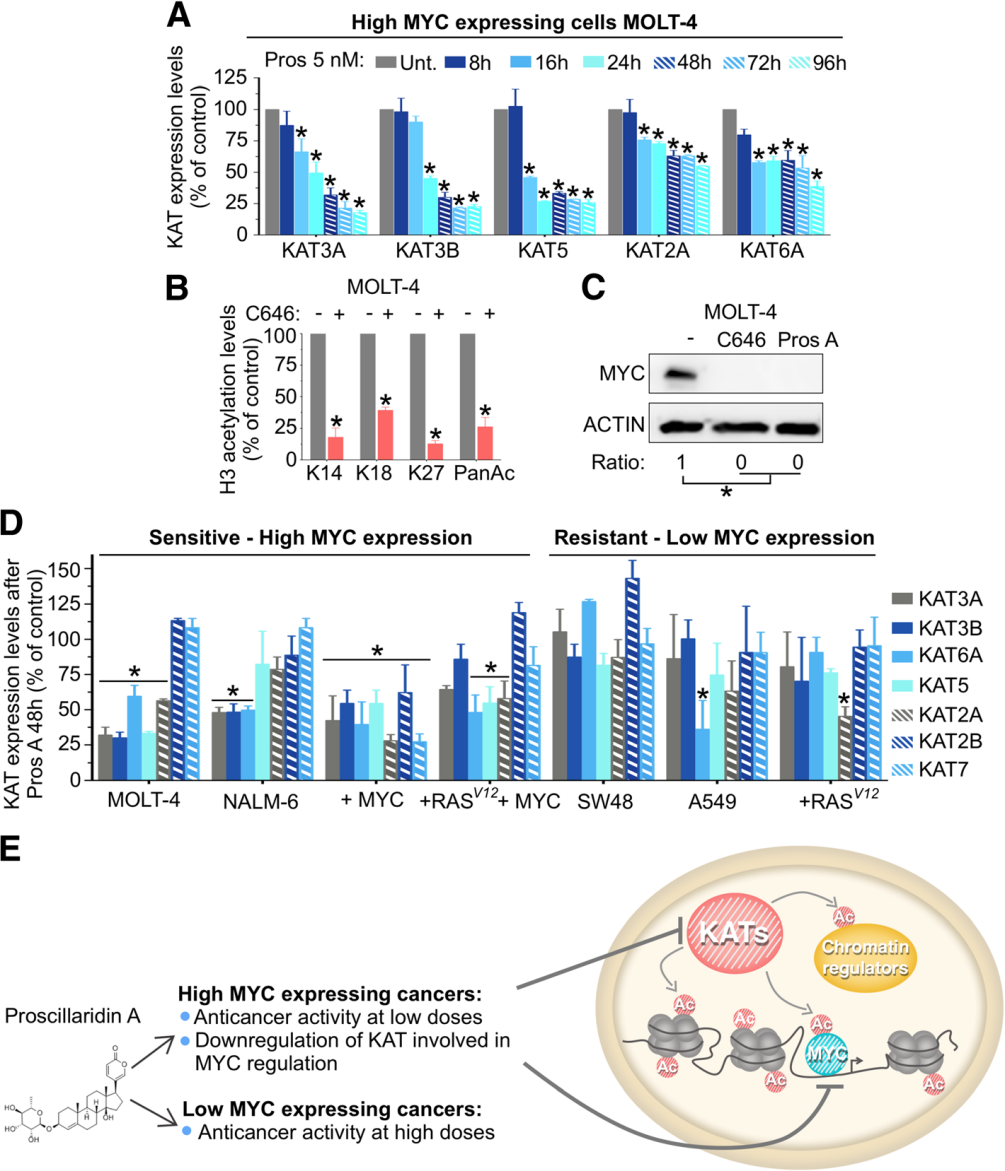
Proscillaridin A Treatment Induces Downregulation of KATs Involved In MYC Acetylation. **a** KAT2A (GCN5), KAT3A (CBP), KAT3B (P300), KAT5 (TIP60), and KAT6A (MOZ) expression levels after proscillaridin A treatment (5 nM, 48 h) were assessed by western blotting in MOLT-4 cells (ACTIN was used as loading control). **b** Quantification of H3 acetylation levels after treatment with KAT3B/A inhibitor C646 (10 μM, 48 h) (* indicates *P* < 0.05; Two-way ANOVA; *n* = 3). **c** MYC protein expression after C646 treatment and proscillaridin A treatment (5 nM; 48 h). ACTIN was used as loading control. **d** MOLT-4, NALM-6, SW48 and A549 cell lines were treated with proscillaridin A (5 nM, 48 h) and fibroblasts transduced with *RAS*^*V12*^, *MYC* and *RAS*^*V12*^/*MYC* were treated with proscillaridin A (70 nM, 48 h). KAT3A (CBP), KAT3B (P300), KAT5 (TIP60), KAT2A (GCN5), KAT2B (PCAF), KAT6A (MOZ) and KAT7 (HBO1) expression levels were assessed by western blotting, quantified and expressed as percentage of untreated cells (* indicates *P* < 0.05; One-way ANOVA; *n* = 3). **e** Scheme showing that proscillaridin A targets high MYC expressing leukemic cells by inhibiting histone acetyltransferases involved in MYC acetylation and stability and causing loss of histone 3 acetylation

We next investigated whether acetylation loss was due to a dysregulation of histone acetyltransferases (KATs). We measured, by western blotting, KAT levels before and after proscillaridin A treatment (5 nM; 8 h–96 h) in MOLT-4 cells. Proscillaridin A produced a time-dependent reduction (up to 80%) of several KATs including KAT3A (CBP), KAT3B (P300), KAT5 (TIP60), KAT2A (GCN5) and KAT6A (MOZ) (Fig. 5a, Additional file 9: Figure S8A). Expression of KAT2B (PCAF) and KAT7 (HBO1) were not altered by the treatment (Additional file 9: Figure S8A and S8B). No significant changes were observed in class I histone deacetylases (HDACs) gene expression, suggesting that acetylation loss mainly involved KATs downregulation (Additional file 9: Figure S8C). Interestingly, KAT downregulation was observed only at the protein level, since proscillaridin A did not significantly decrease their mRNA levels except for KAT2A (Additional file 9: Figure S8D). Significant reduction in KAT protein expression including KAT2A, KAT3A, KAT3B and KAT6A, which target histone H3, occurred 8 h prior to significant H3 acetylation loss [34–36]. Despite KAT5 decrease, a KAT known to acetylate H2A, H3 and H4, no changes in H4 acetylation (total or on specific lysines) were measured after treatment (Additional file 4: Figure S3D) [37]. This result can be explained by the fact that KAT7 (HBO1) expression, which is also involved in H4 acetylation, was not affected by the treatment [38]. To confirm the effects of KAT downregulation in MOLT-4 cells, we used KAT3A/B pharmacological inhibitor C646. Similar to proscillaridin A treatment, C646 (10 μM; 48 h) significantly reduced lysine acetylation (H3K14, H3K18, H3K27, and total H3-acetylation), depleted KATs (KAT3A, and KAT3B) and MYC protein levels (Fig. 5b and c; Additional file 9: Figure S8E and S8F).

Since KATs have overlapping enzymatic activities, we asked if the extent KAT protein downregulation was associated with proscillaridin A sensitivity. We compared KAT protein levels before and after proscillaridin A treatment in *MYC* overexpressing cancer cells (MOLT-4, NALM-6, *MYC* and *RAS*^*V12*^ *+ MYC* transformed fibroblasts) versus low *MYC* expressing cancer cells (SW48, A549, and *RAS*^*V12*^ transformed fibroblasts; Fig. 5d; Additional file 10: Figure S9A). Cancer cell lines were treated at 5 nM for 48 h, which was clinically relevant and close the IC_50_ values of leukemic cells. Transformed fibroblasts were treated at 70 nM for 48 h, which was the IC_50_ value of *MYC* transduced fibroblasts as described in Fig. 1c. After treatment, we observed that KAT protein downregulation was more pronounced in drug-sensitive cells with high *MYC* expression as compared to drug-resistant cells with low *MYC* expression. Indeed, proscillaridin A induced a significant downregulation of 4/7 KATs in MOLT-4 cells, 3/7 KATs in NALM-6 cells, 3/7 KATs in *RAS*^*V12*^ *+ MYC* transformed fibroblasts, and 7/7 KATs in *MYC* transformed fibroblasts (Fig. 5d). Interestingly, downregulated KATs (KAT2A/GCN5, KAT3A/ CBP, KAT3B/P300, KAT5/TIP60 and KAT6A/MOZ) in *MYC* overexpressing cells, were shown to acetylate MYC and increase its stability [33, 39–41]. In stark contrast, proscillaridin A failed to downregulate more than one KATs in low *MYC* expressing cancer cells (SW48, A549 and *RAS*^*V12*^ transformed fibroblasts, Fig. 5d). Thus, proscillaridin A-induced KAT proteins downregulation was more important in high *MYC* expressing cells, which correlated with IC_50_ values within its therapeutic range.

Similar analysis was performed on histone H3 acetylation between high *MYC* expressing cancer cells versus low *MYC* expressing cancer cells (Additional file 10: Figure S9B and S9C). Proscillaridin A induced a significant loss of H3 acetylation in drug-sensitive and *MYC* overexpressing cells (MOLT-4, NALM-6, *MYC* and *RAS*^*V12*^ *+ MYC* transformed fibroblasts). In proscillaridin A-resistant and low expressing *MYC* cancer cells, histone acetylation levels were unchanged in SW48 cells after treatment, which correlated with our previous report (Additional file 9: Figure S8B and S8C) [16]. By contrast, A549 cells lost significantly H3 acetylation after treatment while *RAS*^*V12*^ transformed fibroblasts lost acetylation on some sites (K9, K27 and pan-acetyl) and other sites were not affected (K14 and K18) (Additional file 10: Figure S9B and S9C). These data suggest that proscillaridin A sensitivity is not entirely dependent on histone acetylation loss, suggesting the importance of non-histone acetylation. In summary, proscillaridin A antiproliferative effect was associated with its ability to downregulate simultaneously several KATs resulting in loss of acetylation on histone and non-histone proteins (Fig. 5e).

### Proscillaridin A efficiently targets MYC-driven leukemic stem cell populations

**Fig. 6:**
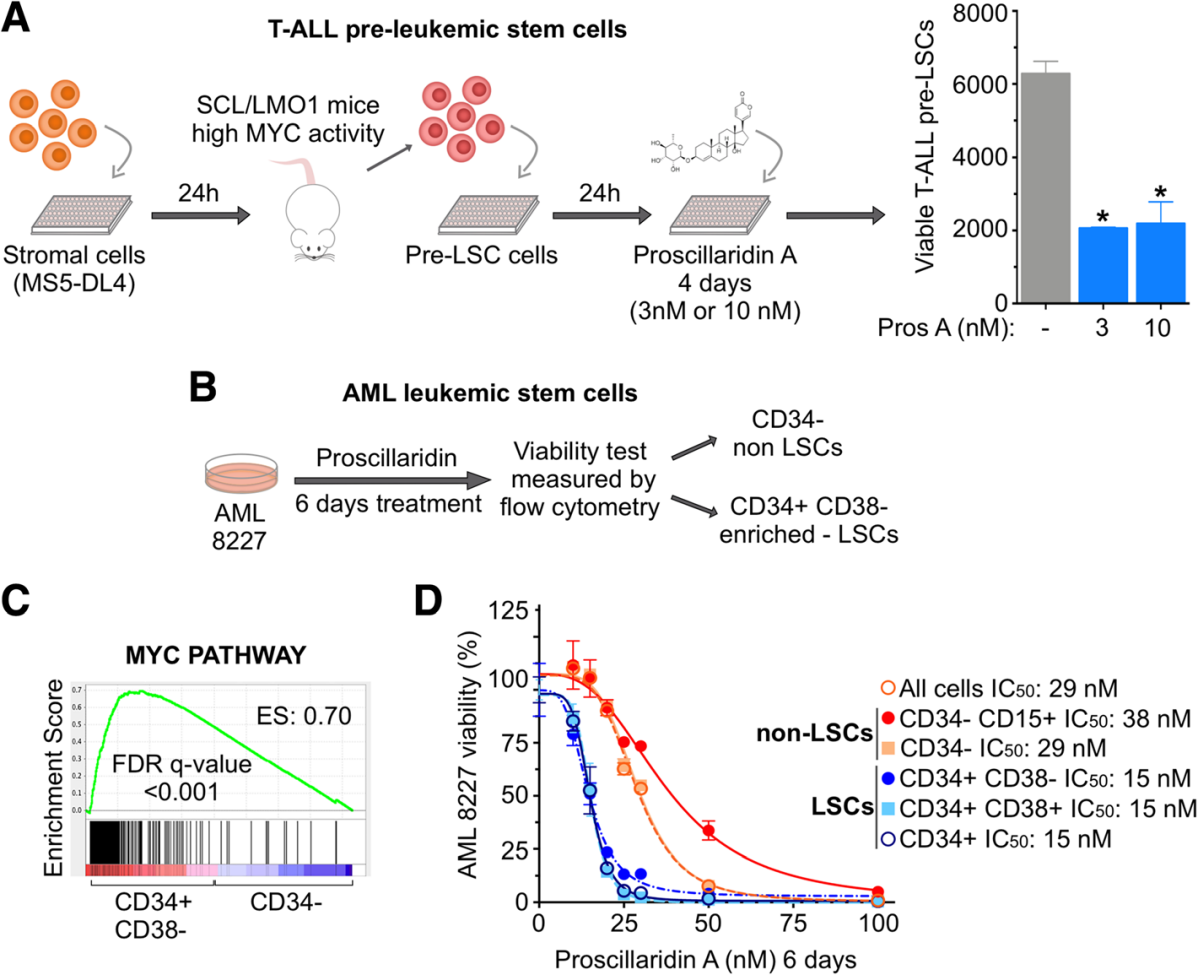
Proscillaridin A Targets Leukemic Stem Cells (LSCs). **a** Cell viability assay of T-ALL pre-LSC co-cultured with MS5-DL4 cells. Proscillaridin A (3 nM or 10 nM) was added 24 h after co-culture, and cells were sorted for pre-LSC viability 4-days post treatment (*indicates *P* < 0.05; One-way ANOVA; *n* ≤ 3). **b** Cell viability assay of AML 8227 population composed of LSCs (CD34^+^) and non-LSCs (CD34^−^/CD15^+/−^). AML 8227 were treated with proscillaridin A (10 nM to 100 nM) for 6 days and cell viability was measured for each cell subgroup by flow cytometry. **c** Gene set enrichment analysis of MYC pathway between two AML 8227 subgroups: LSC-enriched population CD34^+^/CD38^−^ compared to non-LSC population CD34^−^. Enrichment score (ES) and false discovery rate (FDR) rates are shown on the graph. **d** Dose response curves and IC_50_ values after a 6-day proscillaridin A treatment (ranging from 10 nM to 100 nM) in each AML 8227 subgroup (*n* = 3)

We sought to determine whether proscillaridin A could target leukemic stem cells (LSCs) [42, 43]. To explore this possibility, we used two LSC models, a mouse model of T-ALL and a LSC model of human acute myeloid leukemia (AML) [42, 44, 45]. First, pre-LSCs T-ALL cells were isolated from a transgenic mouse model that closely reproduces human T-ALL [46]. We previously showed that these pre-LSCs are driven by the *SCL/TAL1* and *LMO1* oncogenes, which depend on *NOTCH1-MYC* pathways, and are resistant to chemotherapeutic drugs used against leukemia (doxorubicin, camptothecin and dexamethasone) [4, 42]. Low concentrations (3–10 nM) of proscillaridin A significantly decreased pre-LSC T-ALL viability by 70% after 4 days of treatment (Fig. 6a). Despite being resistant to chemotherapeutic drugs, these pre-LSCs (T-ALL) were sensitive to proscillaridin A at clinically relevant doses [42].

Then, we used primary human AML 8227 cells, which contain functional LSCs within the CD34^+^ sub-population, and non-LSC cells characterized by CD34^−^ with or without CD15^+^ expression (Fig. 6b) [44, 45]. Gene set enrichment analysis of data from Lechman et al., revealed that the LSC-enriched fraction (CD34^+^/CD38^−^) in AML 8227 is enriched for MYC target genes expression as compared to non-LSCs (CD34^−^) (Fig. 6c) [44]. After 6 days of proscillaridin A treatment, bulk AML 8227 cells had an IC_50_ of 29 nM (Fig. 6d). Likewise, CD34^−^ with or without CD15^+^ non-LSC cells had IC_50_ of 38 nM and 29 nM, respectively. In contrast, all CD34^+^ AML cells (CD34^+^, CD34^+^/CD38^+^ and CD34^+^/CD38^−^) were more sensitive to proscillaridin A with IC_50_ values of 15 nM. Altogether, proscillaridin A efficiently targets LSC-enriched populations, in both T-ALL and AML models marked by high MYC expression, further supporting its repurposing against MYC-dependent leukemia.

## Discussion

The repurposing potential of cardiac glycosides in oncology has been suggested several decades ago and is currently under intense clinical investigation either alone (in prostate cancer, NCT01162135; breast cancer, NCT01763931; and sarcoma, NCT00017446) or in combination with chemotherapy (digoxin with cisplatin in head and neck cancers, NCT02906800; or with epigenetic drug decitabine, NCT03113071) [47]. However, none of these clinical studies are designed to use cardiac glycosides against cancer types driven by specific oncogene, such as MYC-driven cancers; although the effect of cardiac glycosides on MYC inhibition has been previously reported [48, 49]. Here, we demonstrate that the anticancer activity of proscillaridin A correlates positively with MYC oncogenic expression in leukemia, suggesting its repurposing potential against cancer types defined by a specific molecular signature. Deregulated MYC is found in more than half of hematological malignancies, including T-cell and B-cell neoplasms, lymphomas and myeloid leukemia. Chromosomal translocation, gene amplification, and gene activation contribute to MYC overexpression, which often correlates with a poor prognosis [3, 50]. Interestingly, proscillaridin A exhibited a strong anticancer activity on both lymphoid and myeloid leukemic stem cell populations overexpressing MYC, indicating the potential of controlling leukemia self-renewal capacity.

Proscillaridin A induced a rapid loss of MYC protein expression in MYC-driven leukemia cells through the downregulation of series of KATs (KAT2A, KAT3A, KAT3B, KAT5 and KAT6A) known to be involved in MYC protein stability (by lysine acetylation) and activation of MYC transcriptional programs [33, 39–41, 51]. Interestingly, KAT downregulation induced by proscillaridin A was associated with a significant reduction of MYC protein half-life. Since KATs have overlapping activity and targets, it may be relevant to target these enzymes simultaneously to ensure an efficient reduction in lysine acetylation of MYC and its partners [12, 39, 41, 51]. This may represent an alternative strategy as opposed to pharmacological inhibition of a specific KAT to overcome the redundant activity of KATs [52].

Proscillaridin A treatment also resulted in a significant loss of H3 acetylation levels in MYC overexpressing cells. This effect was associated with the loss of epigenetic active marks (including H3K27ac) in promoters of MYC-dependent genes associated with cell proliferation programs. Interestingly, MYC-driven leukemic cells started to differentiate during treatment and after drug removal, suggesting the onset of a stable epigenetic reprogramming. The contribution of global histone acetylation loss to the anticancer effects of proscillaridin A remains to be better understood since loss of histone acetylation was observed in some proscillaridin A-resistant cells, suggesting that loss of H3 acetylation may not be sufficient to modulate cell viability in these low MYC expressing cells.

## Conclusion

Here, we demonstrated that proscillaridin A treatment in MYC overexpressing leukemia cells, led to the downregulation of several KATs, induced significant acetylation loss, produced MYC degradation, and induced persistent epigenetic effects and leukemia cell differentiation, which was maintained for several days after drug removal. Therefore, this study supports a strategy of simultaneously targeting several KATs to reduce efficiently acetylation in histone and in non-histone proteins, to overcome the redundant activity of KATs. The mechanism implicated in proscillaridin A-induced KATs downregulation is under investigation. Overall, we conclude that proscillaridin A has a promising potential to be repurposed as an epigenetic drug in personalized oncology, particularly in MYC overexpressing leukemia.

## Additional files

Additional file 1: Supplementary Materials and Methods. (DOCX 24 kb)

Additional file 2: **Figure S1.** MYC Expression Correlates with Proscillaridin A Anticancer Efficacy. **A** Upper panel, half maximal inhibitory concentration (IC_50_) after a 24h proscillaridin A treatment (ranging from 1 nM to 100 μM) in a panel of human cancer cell lines (n=4). Lower panel, MYC protein level in each untreated cell line, assessed by western blotting. ACTIN was used as a loading control (n ≥ 3). **B** Graph showing MYC expression (relative to ACTIN) compared to proscillaridin A IC_50_ (24h) in 14 cancer cell lines. Correlation was evaluated by linear regression analysis; P-value is shown on the graph (n=3). **C** Representative pictures of transformed primary human fibroblasts before and after transduction with *RAS*^*V12*^, *MYC* and *RAS*^V12^/*MYC* were taken by light microscopy (400X magnification). **D** and **E** MYC expression was assessed by Western blotting in WT and MYC-transduced MOLT-4 cells (**D**) and REH cells (**E**). MYC expression was calculated as a ratio over ACTIN levels (*indicates P<0.05; One-way ANOVA; n = 3). IC_50_ values after 24h proscillaridin A treatment (ranging from 0.1 nM to 1 μM) in MOLT-4 cells (**D**) and REH cells (**E**) (n ≥ 3). **F** Time course experiment in NALM-6 cells treated with 5 nM for up to 96h. MYC expression was calculated as a ratio over ACTIN levels (*indicates P<0.05; One-way ANOVA; n = 3). (PDF 1640 kb)

Additional file 3: **Figure S2.** Transcriptomic Analysis In MOLT-4 Cells Treated with Proscillaridin A (5 nM, 48h). **A** Heat map representing RPKM similarities between triplicates of untreated (U) and Proscillaridin A-treated (5 nM; 48h; T) MOLT-4 cells (n = 3). Red color corresponds to the highest similarity and yellow corresponds to the lowest similarity. **B** Proscillaridin A (5 nM, 48h) induced gene expression reprogramming of MOLT-4 cells. Volcano plots of gene expression changes in MOLT-4 cells in untreated versus treated samples. Black dots correspond to genes with P-value adjusted > 0.5. Grey dots correspond to genes with P-value adjusted < 0.5 but without significant fold change expression difference between untreated and treated cells (-0.5 < FC < 1). Downregulated genes with P-value adjusted < 0.5 and FC < -0.5 are shown in green. Upregulated genes with P-value adjusted < 0.5 and FC > 1 are shown in red. Numbers of downregulated and upregulated genes are shown on the graphs. **C** Metascape analysis of genes downregulated by proscillaridin A treatment (5 nM; 48h). **D** Cell cycle analysis after BrdU staining in MOLT-4 and NALM-6 cell lines exposed to proscillaridin A (5 nM, 48h). Cell fluorescence was measured by flow cytometry (* indicates P<0.05; Two-way ANOVA; n=3). **E** Metascape analysis of genes upregulated by proscillaridin A treatment (5 nM; 48h). (PDF 905 kb)

Additional file 4: **Figure S3.** Proscillaridin A Induced Histone 3 Acetylation Loss In MOLT-4 And NALM-6 Cells. **A** MOLT-4 cells were treated with proscillaridin A (5 nM) and histones were acid-extracted after 8, 16, 24, 48, 72 and 96 hours. H3 acetylation levels were quantified and expressed as a percentage of untreated cells (* indicates P<0.05; Two-way ANOVA; n = 3). **B** Ratio of chromatin immunoprecipitation (ChIP) of H3K27 acetylation in MOLT-4 cells before and after proscillaridin A treatment (5 nM; 48h) (*indicates P<0.001; paired t-test, n=3). **C** NALM-6 cells were treated with proscillaridin A (5 nM) and histones were acid-extracted after 8, 16, 24, 48, 72 and 96 hours. H3 acetylation levels were quantified and expressed as a percentage of untreated cells (* indicates P<0.05; Two-way ANOVA; n = 3). **D** MOLT-4 and **E** NALM-6 cells were treated with proscillaridin A (5 nM) and histones were acid-extracted after 8, 16, 24, 48, 72 and 96 hours. Histone 4 acetylation levels were assessed using antibodies against K5ac, K8ac, K16ac, K20ac, and total histone 4 acetylation. H4 was used as loading control. H4 acetylation levels were quantified and expressed as a percentage of untreated cells (* indicates P<0.05; Two-way ANOVA; n = 3). (PDF 567 kb)

Additional file 5: **Figure S4.** Histone Methylation Is Not Significantly Altered After Proscillaridin A Treatment On Histone H3. MOLT-4 (**A**) and NALM-6 (**B**) cells were treated with proscillaridin A (5 nM) and histones were acid-extracted after 8, 16, 24, 48, 72 and 96 hours. Histone 3 methylation levels were assessed using antibodies against K4me3, K9me3, and K27me3. H3 was used as loading control. H3 methylation levels were quantified and expressed as a percentage of untreated cells (Two-way ANOVA; n = 3). **C** Confocal microscopy (60X) of MOLT-4 cells stained with DAPI revealed heterochromatin modulation after proscillaridin A treatment (5 nM; 48h). White arrows indicate loss of heterochromatin regions. (PDF 1592 kb)

Additional file 6: **Figure S5.** H3K27 Acetylation DNA Occupancy Is Lost After Proscillaridin A Treatment In MOLT-4 Cells. Metascape analysis of **A** downregulated genes and **B** upregulated genes after proscillaridin A treatment (5 nM; 48h) marked by H3K27ac in their promoter regions (-500 bp / +500 bp). (PDF 657 kb)

Additional file 7: **Figure S6.** Proscillaridin A Treatment Downregulated MYC Target Genes That Are Marked By H3K27ac In Promoter Regions. Map of **A** co-expression pathways and **B** protein-protein physical interactions of MYC target genes marked by H3K27ac in untreated MOLT-4 cells. (PDF 1418 kb)

Additional file 8: **Figure S7.** Loss of Acetylation In MYC Protein And MYC After Proscillaridin A Treatment In High MYC Expressing Cells. **A** Mass spectrometry analysis on 2 MYC peptides (LVSEK(ac)LASYQAAR) after proscillaridin A treatment (5nM; 48h) in MOLT-4. Log_2_ normalized intensity is shown (* indicates P<0.001; paired t-test; n=4). **B** Map of co-expression pathways of the 28 proteins that lost acetylation after proscillaridin A treatment (5 nM; 48h) in MOLT-4 cells. (PDF 890 kb)

Additional file 9: **Figure S8.** MYC Inhibition Induced By Proscillaridin A Is Regulated By KAT Activities. **A** MOLT-4 cells were treated with proscillaridin A (5 nM) and KAT3A, KAT3B, KAT5, KAT2A, KAT2B, KAT6A and KAT7 expression levels were assessed by western blotting. ACTIN was used as loading control. **B** KAT2B and KAT7 expression levels were quantified and expressed as percentage of untreated cells (n=3). **C and D** Class I HDAC (**C**) and KAT (**D**) expression transcripts (RPKM) expression after proscillaridin A treatment (5 nM; 48h) in RNA-sequencing data set (*indicates Log_2_ FC<-0.5 and $ indicates Log_2_ FC > 1). **E** MOLT-4 cells were treated with KAT3B/A inhibitor C646 (10 μM) and with proscillaridin A (5 nM) and histones were acid-extracted after 48 hours. Histone 3 acetylation levels were assessed using antibodies against K14ac, K18ac, K27ac, and pan histone 3 acetylation. H3 was used as loading control. **F** Left panel, MOLT-4 cells were treated with KAT3B/A inhibitor C646 (10 μM) and with Proscillaridin A (5 nM) and KAT5, KAT3A and KAT3B expression levels were assessed by western blotting. ACTIN was used as loading control. Right panel, KAT5, KAT3A and KAT3B levels were quantified and expressed as a percentage of untreated cells (* indicates P<0.05; Two-way ANOVA; n = 3). (PDF 1077 kb)

Additional file 10: **Figure S9.** Proscillaridin A Induces KAT Downregulation Specifically In High MYC Expressing Cells. **A-C** MOLT-4, NALM-6, SW48 and A549 cell lines were treated with proscillaridin A (5 nM, 48h) and fibroblasts transduced with *RAS*^*V12*^, *MYC* and *RAS*^*V12*^*/MYC* were treated with proscillaridin A (5 nM or 70 nM, 48h). **A** KAT3A (CBP), KAT3B (P300), KAT5 (TIP60), KAT2A (GCN5), KAT2B (PCAF), KAT6A (MOZ) and KAT7 (HBO1) expression levels were assessed by western blotting. ACTIN was used as loading control. **B** Histone 3 acetylation levels were assessed by using antibodies against K9ac, K14ac, K18ac, K27ac, and pan histone 3 acetylation. H3 total was used as loading control. **C** Histone 3 acetylation levels were quantified and expressed as percentage of control (* indicates P<0.05; One-way ANOVA; n=3). (PDF 2285 kb)

## Data Availability

The datasets used and/or analysed during the current study are available from the corresponding author on reasonable request.

## Abbreviations

AML: Acute myeloid leukemia
H3: Histone 3
H4: Histone 4
HDAC: Histone deacetylase
IC_50_: Half-maximal inhibitory concentration
KAT: Lysine acetyltransferase
LSCs: Leukemic stem cells
MYC: c-MYC
qPCR: quantitative RT-PCR
T-ALL: T-cell acute lymphoblastic leukemia
TPA: 12-O-Tetradecanoylphorbol-13-acetate
