# Supplementary material for: Heart failure drug proscillaridin A targets MYC overexpressing leukemia through global loss of lysine acetylation
Source: J Exp Clin Cancer Res. 2019 Jun 13;38:251. doi: 10.1186/s13046-019-1242-8 (PMC6563382; doi:10.1186/s13046-019-1242-8)
Supplement: Supplementary file 1 — Supplementary Materials and Methods. (DOCX 24 kb) [file 13046_2019_1242_MOESM1_ESM.docx]

**Supplementary Materials and Methods**

**Cell Culture and Drug Treatments**

MOLT-4 (T-cell acute lymphoblastic leukemia), NALM-6 (pre-B-cell acute lymphoblastic leukemia), REH (pre-B acute lymphoblastic leukemia), KOPN-8 (B-cell precursor acute lymphoblastic leukemia), CCRF-CEM (T-cell acute lymphoblastic leukemia), K562 (chronic myelogenous leukemia) and DLD-1 (colorectal-adenocarcinoma) cells were cultured in RPMI-1640 (Life Technologies, USA) supplemented with 10% fetal bovine serum (FBS, Wisent Inc., Canada). CALU-6 (anaplastic carcinoma) cells were cultured in MEM (Life Technologies, USA). RD (embryonal rhabdomyosarcoma) and MCF7 (breast adenocarcinoma) cells were cultured in DMEM (Life Technologies, USA) supplemented with 10% FBS. U2OS (osteosarcoma) cells were cultured in McCoy’s (Life Technologies, United Kingdom) supplemented with 10% FBS. A549 (lung carcinoma) cells were cultured in F12K (GE Healthcare Life Sciences, Canada) supplemented with 10% of FBS. SW48 (colorectal adenocarcinoma) cells were cultured in Leibovitz L-15 supplemented with 10% FBS. Cell lines were purchased from American Type Culture Collection and tested for mycoplasma contamination. All cell lines were culture in a humidified incubator with 5% CO_2_ at 37°C, except SW48 cells that were cultured with 1% CO_2_. K562 cells were provided by Dr Elie Haddad and MCF7 cells were supplied by Dr Audrey Claing. AML 8227 were cultured in StemSpan^TM^ SFEM II (STEMCELL Technologies) supplemented with growth factors (Life Technologies: 10 ng/mL IL3, IL6 and G-CSF, 25 ng/mL TPO, 50 ng/mL SCF and FLT3L, and 100 ng/mL SCF) and penicillin-streptomycin (Life Technologies). Phorbol 12-myristate 13-acetate (TPA) was purchased from Sigma-Aldrich, and C646 (Histone Acetyltransferase KAT3B/KAT3A Inhibitor) was purchased from EMD Millipore.

**Growth Inhibition**

ORFLO MOXI Mini Automated Cell Counter was used to analyze growth inhibition after 48h treatment. IC_50_ values were calculated with GraphPad Prism software. GUAVA EasyCyte (Millipore Sigma-Aldrich) was used to measure cell viability after 24h treatment. Viacount reagent (Millipore Sigma-Aldrich cat#4000-0040) was added prior to analysis. For AML 8227 cells, proscillaridin A was added at specified concentrations and incubated for 6 days. Cells were analyzed by flow cytometry. Phenotype and viability were assessed using CD34-APC (581), CD38-PE (HB-7), CD15-FITC (HI98) and SYTOX Blue (Life Technologies). All antibodies were purchased from BioLegend. Flow cytometry was performed using a LSRFortessa fitted with a high-throughput sampler (BD Biosciences).

**Nuclei Imaging**

Cell pellets were fixed for 10 minutes at room temperature with 3.7% formaldehyde (Anachemia, cat#41883-360) and then permeabilized for 10 minutes at room temperature with 0.1% triton (PBS). Nuclei were stained and mounted with ProLong™ Diamond Antifade Mountant with DAPI (Invitrogen; cat#36962). Images were acquired using Leica TCS SP8 confocal microscope.

**Cell Cycle Analysis**

Cells were treated with proscillaridin A for 48 hours at 3 different concentrations (2.5, 5, or 7.5 nM). Cells were then harvested, fixed, and stained with bromodeoxyuridine (BrdU) and/or 7-amino-actinomycin (7-AAD) (BD Pharmingen BrdU Flow Kit). Samples were analyzed by Fluorescence-activated Cell Sorting (FACS) on a BD FACS Canto II.

**T-cell differentiation assay**

GUAVA EasyCyte (Millipore Sigma-Aldrich) was used to measure T-cells differentiation on MOLT-4 cells. TCR expression was measured by staining MOLT-4 cells with APC IGM $\kappa$ Isotype Control (BD pharmigen; cat#550883APC) and TCR alpha beta (BD pharmigen, cat#563826) and IGG. CD3 expression was measured by staining MOLT-4 cells with PE-Cy^tm^ IGG1 $\kappa$ isotype (BD pharmigen, cat#557872) and PE-Cy^tm^ CD3 (BD pharmigen, cat#560910).

**Antibodies for western blots**

The following primary antibodies were used to detect expression levels of isolated proteins: C-MYC (1:5000 Abcam cat#AB32072), RAS (1:2500 Millipore HA-RAS clone MC57, cat#ng1905692); KAT3A (1:1000 Cell Signaling cat#7389), KAT3B (1:2000 Active Motif cat#61402), KAT2A (1:1000 Santa Cruz cat#sc-365321), KAT2B (1:1000 Cell Signaling cat#3378), KAT5 (1:2500 Abcam cat#ab137518), KAT6A (1:1000 Active Motif cat#39868), KAT7 (1:1000 Bethyl Labs cat#A302-224A-T); and Actin (1:5000 Sigma-Aldrich cat#A2228).

The following primary antibodies were used to detect histone acetylation marks on H3 and H4 subunits: H3K9ac (1:5000 Active Motif cat#39917), H3K14ac (1:5000 Active Motif cat#39698), H3K18ac (1:2500 Active Motif #39588), H3K27ac (1:5000 Active Motif #39134), H3ac (pan-acetyl) (1:5000 Active Motif cat#39140), H3 (1:5000 Active Motif cat#39763), H4K5ac (1:2000 Active Motif cat#39700), H4K8ac (1:5000 Active Motif cat#39172); H4K12ac (1:5000 Active Motif cat#39928), H4K16ac (1:5000 Active Motif cat#39167), H4K20ac (1:2000 Active Motif cat#61531), H4ac (pan-acetyl) (1:5000 Active Motif cat#39244) and H4 (1:5000 Active Motif cat#39270).

**Acetylome analysis by mass spectrometry**

Sample preparation: Pellets were resuspended in 1 ml of lysis buffer (8 M Urea, 0.1 M Tris-HCl pH 8.0, 150 mM NaCl, 10 mM sodium butyrate (Sigma 303410), 10 mM nicotinamide (Sigma 3376), with Roche protease inhibitor tablet w/o EDTA and lysed using 5 passes through a 21-gauge needle followed by clarification at 15,000 g for 10 minutes at room temperature. The supernatant was quantitated using a Pierce BCA protein kit. One mg of protein was reduced with TCEP (Sigma C4706–2, 4 mM final concentration), alkylated with iodoacetamide (Sigma L1149–5G, prepared fresh in water at 10 mM), and quenched with 10 mM DTT. Samples were diluted to < 2 M urea with 0.1 M Tris-HCl pH 8.0 prior to overnight digestion with trypsin (Pierce 90058, in 50 mM acetic acid, ~15 µg/sample) at room temperature.

Digested samples were acidified using 10% Trifluoroacetic acid (TFA) to a final pH of < 3. Subsequently, insoluble material was precipitated by centrifugation at 10,000 g at room temperature. Clarified supernatant was loaded onto a Sep Pak tC18 column (Waters) activated with 1 ml 80% ACN/0.1% TFA and equilibrated with 3 × 1 ml 0.1% TFA. The columns were washed with 5 × 1 ml 0.1% TFA and eluted with 1 ml 40% ACN/0.1% TFA. The eluted material was frozen and lyophilized over 48 h to completely remove TFA. Dried peptides were resuspended in 800 µl IP buffer (0.1 M Tris-HCl pH 8, 50 mM NaCl, Roche protease inhibitor tablet w/o EDTA) and clarified by centrifugation for 2 minutes at 10,000 g. For purification of acetylated peptides, supernatant was incubated with 50 µl packed volume of ImmuneChem anti-acetyl-lysine agarose beads (ICP0388). Immunoprecipitation was carried out at 4°C for 2 hours. Beads were washed twice with 1 ml lysis buffer and twice with 1 ml water prior to elution with 0.15% TFA at room temperature. Two elutions of 60 µl (10 minutes each) were pooled for analysis.

Mass spectrometry set up: In preparation for mass spectrometry, samples were loaded onto Pierce C18 spin tips (84850) previously activated with 0.1% TFA/80% acetonitrile (ACN) and washed with 0.1% TFA. Following application of the sample, tips were washed twice with 0.1 % TFA and eluted with 0.1% TFA/40% ACN. The elutions were dried on a speed-vac without heat for 4 hours. Acetyl-lysine-enriched peptides were run in technical duplicate on a Thermo Fisher Orbitrap Fusion mass spectrometry system. This system is equipped with an Easy nLC 1200 ultra-high pressure liquid chromatography system interfaced via a Nanospray Flex nanoelectrospray source. The samples were injected on a C18 reverse phase column (25 cm x 75 um packed with ReprosilPur C18 AQ 1.9 µm particles). An organic gradient from 5% to 30% ACN in 0.1% formic acid over 112 minutes at a flow rate of 300 nl/min was used to separate peptides. Spectra were continuously collected by the MS in a data-dependent manner throughout the gradient, acquiring a full scan in the Orbitrap (at 120,000 resolution with an AGC target of 200,000 and a maximum injection time of 100 ms) followed by as many MS/MS scans as could be acquired on the most abundant ions in 3s in the dual linear ion trap. A rapid scan type with an intensity threshold of 5000, HCD collision energy of 29%, AGC target of 10,000, a maximum injection time of 35 ms, and an isolation width of 1.6 m/z was used. All singly and unassigned charge states were rejected. Dynamic exclusion was enabled with a repeat count of 1. The exclusion duration used was 20 s with an exclusion mass width of +/- 10 ppm.

Modification and determination of changes: Variable modifications were allowed for N-terminal protein acetylation, methionine oxidation, and lysine acetylation. A static modification was indicated for carbamidomethyl cysteine. All other settings were left as default in the MaxQuant program (PMID: 19029910; version 1.5.5.1; *H. sapiens* database downloaded on January 11 2016). Decoy hits, contaminants and peptides that did not contain acetyl-lysine residues were removed. All samples were normalized across fractions by median-centering the log2-transformed MS1-intensity distributions. MSstats group comparison function was run with MSstats Bioconductor package (version 3.3.10) and the following options: no interaction terms for missing values, no interference, unequal intensity feature variance, restricted technical and biological scope of replication (PMID: 24794931). Statistically significant changing proteins were selected by applying a log2-fold-change (>1.0) and an adjusted p-value (< 0.05) corrected for multiple testing threshold.

UniProtKB identification and acetylation sites analyzed on Figure 4 (4D and 4E) are: ENO1 (P06733_ac126); HNRNPC (P07910_ac50); MIF (P14174_ac78); MYC (P01106_ac148); NPM1 (P06748_ac273); PTMA (P06454_ac1_ac18); TCF12 (Q99081_ac151); HNRNPU (Q00839_ac565); BRPF1 (P55201_ac896); H2AFV (Q71UI9_ac5_ac8); JADE2 (Q9NQC1_ac32_ac38); JADE3 (Q92613_ac735); NCOR2 (Q9Y618_ac2037); RBBP4 (Q09028_ac1_ac4).

**Supplementary Figures:**

**Supplemental Figure S1.** MYC Expression Correlates with Proscillaridin A Anticancer Efficacy. **A** Upper panel, half maximal inhibitory concentration (IC_50_) after a 24h proscillaridin A treatment (ranging from 1 nM to 100 µM) in a panel of human cancer cell lines (n=4). Lower panel, MYC protein level in each untreated cell line, assessed by western blotting. ACTIN was used as a loading control (n ≥ 3). **B** Graph showing MYC expression (relative to ACTIN) compared to proscillaridin A IC_50_ (24h) in 14 cancer cell lines. Correlation was evaluated by linear regression analysis; P-value is shown on the graph (n=3). **C** Representative pictures of transformed primary human fibroblasts before and after transduction with *RAS^V12^*, *MYC* and *RAS*^V12^/*MYC* were taken by light microscopy (400X magnification). **D** and **E** MYC expression was assessed by Western blotting in WT and MYC-transduced MOLT-4 cells (**D**) and REH cells (**E**). MYC expression was calculated as a ratio over ACTIN levels (*indicates P<0.05; One-way ANOVA; n = 3). IC_50_ values after 24h proscillaridin A treatment (ranging from 0.1 nM to 1 µM) in MOLT-4 cells (**D**) and REH cells (**E**) (n ≥ 3). **F** Time course experiment in NALM-6 cells treated with 5 nM for up to 96h. MYC expression was calculated as a ratio over ACTIN levels (*indicates P<0.05; One-way ANOVA; n = 3).

**Supplemental Figure S2.** Transcriptomic Analysis In MOLT-4 Cells Treated with Proscillaridin A (5 nM, 48h). **A** Heat map representing RPKM similarities between triplicates of untreated (U) and Proscillaridin A-treated (5 nM; 48h; T) MOLT-4 cells (n = 3). Red color corresponds to the highest similarity and yellow corresponds to the lowest similarity. **B** Proscillaridin A (5 nM, 48h) induced gene expression reprogramming of MOLT-4 cells. Volcano plots of gene expression changes in MOLT-4 cells in untreated versus treated samples. Black dots correspond to genes with P-value adjusted > 0.5. Grey dots correspond to genes with P-value adjusted < 0.5 but without significant fold change expression difference between untreated and treated cells (-0.5 < FC < 1). Downregulated genes with P-value adjusted < 0.5 and FC < -0.5 are shown in green. Upregulated genes with P-value adjusted < 0.5 and FC > 1 are shown in red. Numbers of downregulated and upregulated genes are shown on the graphs. **C** Metascape analysis of genes downregulated by proscillaridin A treatment (5 nM; 48h). **D** Cell cycle analysis after BrdU staining in MOLT-4 and NALM-6 cell lines exposed to proscillaridin A (5 nM, 48h). Cell fluorescence was measured by flow cytometry (* indicates P<0.05; Two-way ANOVA; n=3). **E** Metascape analysis of genes upregulated by proscillaridin A treatment (5 nM; 48h).

**Supplemental Figure S3.** Proscillaridin A Induced Histone 3 Acetylation Loss In MOLT-4 And NALM-6 Cells. **A** MOLT-4 cells were treated with proscillaridin A (5 nM) and histones were acid-extracted after 8, 16, 24, 48, 72 and 96 hours. H3 acetylation levels were quantified and expressed as a percentage of untreated cells (* indicates P<0.05; Two-way ANOVA; n = 3). **B** Ratio of chromatin immunoprecipitation (ChIP) of H3K27 acetylation in MOLT-4 cells before and after proscillaridin A treatment (5 nM; 48h) (*indicates P<0.001; paired t-test, n=3). **C** NALM-6 cells were treated with proscillaridin A (5 nM) and histones were acid-extracted after 8, 16, 24, 48, 72 and 96 hours. H3 acetylation levels were quantified and expressed as a percentage of untreated cells (* indicates P<0.05; Two-way ANOVA; n = 3). **D** MOLT-4 and **E** NALM-6 cells were treated with proscillaridin A (5 nM) and histones were acid-extracted after 8, 16, 24, 48, 72 and 96 hours. Histone 4 acetylation levels were assessed using antibodies against K5ac, K8ac, K16ac, K20ac, and total histone 4 acetylation. H4 was used as loading control. H4 acetylation levels were quantified and expressed as a percentage of untreated cells (* indicates P<0.05; Two-way ANOVA; n = 3).

**Supplemental Figure S4.** Histone Methylation Is Not Significantly Altered After Proscillaridin A Treatment On Histone H3. MOLT-4 (**A**) and NALM-6 (**B**) cells were treated with proscillaridin A (5 nM) and histones were acid-extracted after 8, 16, 24, 48, 72 and 96 hours. Histone 3 methylation levels were assessed using antibodies against K4me3, K9me3, and K27me3. H3 was used as loading control. H3 methylation levels were quantified and expressed as a percentage of untreated cells (Two-way ANOVA; n = 3). **C** Confocal microscopy (60X) of MOLT-4 cells stained with DAPI revealed heterochromatin modulation after proscillaridin A treatment (5 nM; 48h). White arrows indicate loss of heterochromatin regions.

**Supplemental Figure S5.** H3K27 Acetylation DNA Occupancy Is Lost After Proscillaridin A Treatment In MOLT-4 Cells. Metascape analysis of **A** downregulated genes and **B** upregulated genes after proscillaridin A treatment (5 nM; 48h) marked by H3K27ac in their promoter regions (-500 bp / +500 bp).

**Supplemental Figure S6.** Proscillaridin A Treatment Downregulated MYC Target Genes That Are Marked By H3K27ac In Promoter Regions. Map of **A** co-expression pathways and **B** protein-protein physical interactions of MYC target genes marked by H3K27ac in untreated MOLT-4 cells.

**Supplemental Figure S7.** Loss of Acetylation In MYC Protein And MYC After Proscillaridin A Treatment In High MYC Expressing Cells. **A** Mass spectrometry analysis on 2 MYC peptides (LVSEK(ac)LASYQAAR) after proscillaridin A treatment (5nM; 48h) in MOLT-4. Log_2_ normalized intensity is shown (* indicates P<0.001; paired t-test; n=4). **B** Map of co-expression pathways of the 28 proteins that lost acetylation after proscillaridin A treatment (5 nM; 48h) in MOLT-4 cells.

**Supplemental Figure S8.** MYC Inhibition Induced By Proscillaridin A Is Regulated By KAT Activities. **A** MOLT-4 cells were treated with proscillaridin A (5 nM) and KAT3A, KAT3B, KAT5, KAT2A, KAT2B, KAT6A and KAT7 expression levels were assessed by western blotting. ACTIN was used as loading control. **B** KAT2B and KAT7 expression levels were quantified and expressed as percentage of untreated cells (n=3). **C and D** Class I HDAC (**C**) and KAT (**D**) expression transcripts (RPKM) expression after proscillaridin A treatment (5 nM; 48h) in RNA-sequencing data set (*indicates Log_2_ FC<-0.5 and $ indicates Log_2_ FC > 1). **E** MOLT-4 cells were treated with KAT3B/A inhibitor C646 (10 µM) and with proscillaridin A (5 nM) and histones were acid-extracted after 48 hours. Histone 3 acetylation levels were assessed using antibodies against K14ac, K18ac, K27ac, and pan histone 3 acetylation. H3 was used as loading control. **F** Left panel, MOLT-4 cells were treated with KAT3B/A inhibitor C646 (10 µM) and with Proscillaridin A (5 nM) and KAT5, KAT3A and KAT3B expression levels were assessed by western blotting. ACTIN was used as loading control. Right panel, KAT5, KAT3A and KAT3B levels were quantified and expressed as a percentage of untreated cells (* indicates P<0.05; Two-way ANOVA; n = 3).

**Supplemental Figure S9.** Proscillaridin A Induces KAT Downregulation Specifically In High MYC Expressing Cells. **A-C** MOLT-4, NALM-6, SW48 and A549 cell lines were treated with proscillaridin A (5 nM, 48h) and fibroblasts transduced with *RAS^V12^*, *MYC* and *RAS^V12^/MYC* were treated with proscillaridin A (5 nM or 70 nM, 48h). **A** KAT3A (CBP), KAT3B (P300), KAT5 (TIP60), KAT2A (GCN5), KAT2B (PCAF), KAT6A (MOZ) and KAT7 (HBO1) expression levels were assessed by western blotting. ACTIN was used as loading control. **B** Histone 3 acetylation levels were assessed by using antibodies against K9ac, K14ac, K18ac, K27ac, and pan histone 3 acetylation. H3 total was used as loading control. **C** Histone 3 acetylation levels were quantified and expressed as percentage of control (* indicates P<0.05; One-way ANOVA; n=3).
