# Supplementary figures and images for: Heart failure drug proscillaridin A targets MYC overexpressing leukemia through global loss of lysine acetylation
Source: J Exp Clin Cancer Res. 2019 Jun 13;38:251. doi: 10.1186/s13046-019-1242-8 (PMC6563382; doi:10.1186/s13046-019-1242-8)

**Figure S1**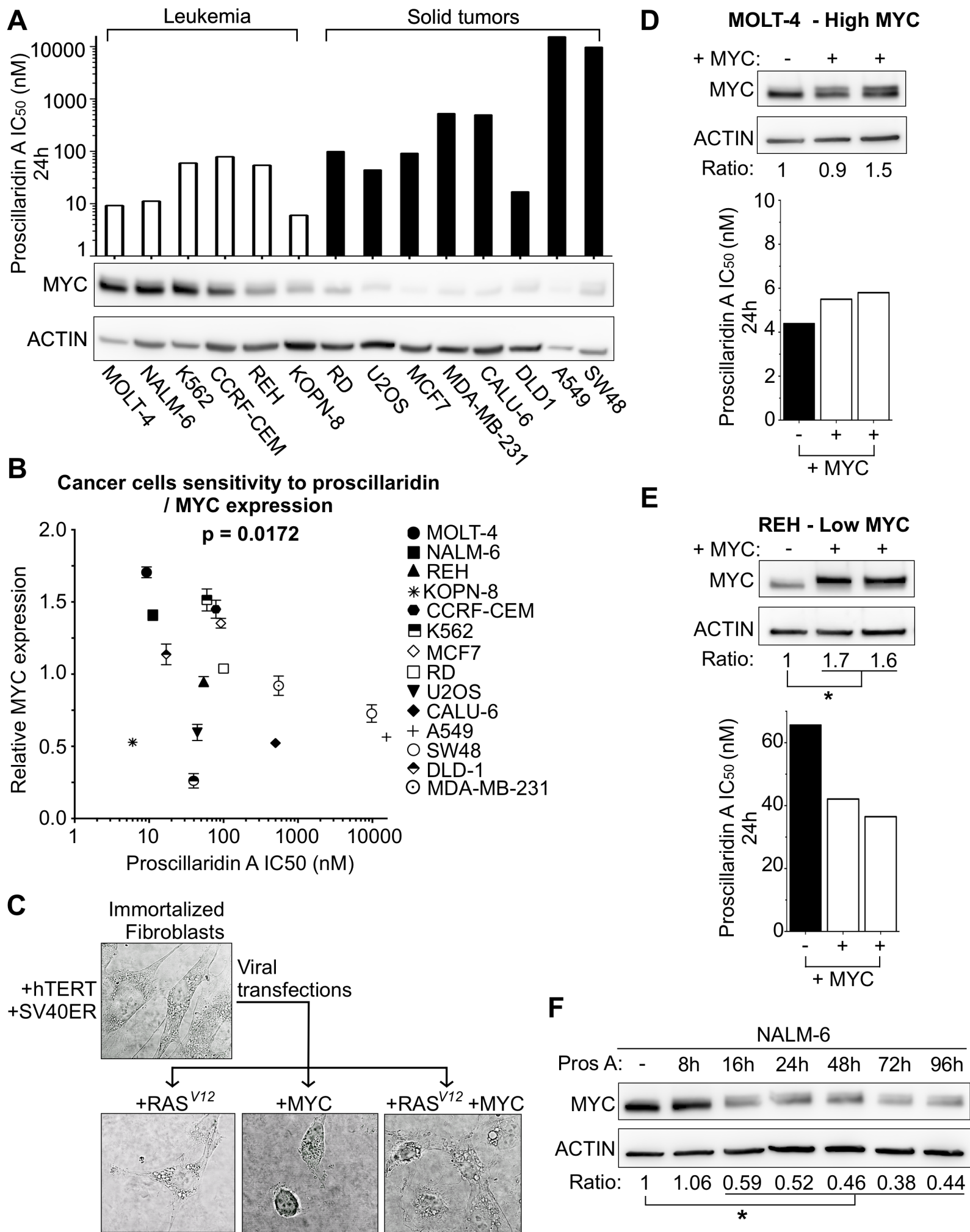

Supplement: Supplementary file 2 — Figure S1. MYC Expression Correlates with Proscillaridin A Anticancer Efficacy. A Upper panel, half maximal inhibitory concentration (IC50) after a 24h proscillaridin A treatment (ranging from 1 nM to 100 μM) in a panel of human cancer cell lines (n=4). Lower panel, MYC protein level in each untreated cell line, assessed by western blotting. ACTIN was used as a loading control (n ≥ 3). B Graph showing MYC expression (relative to ACTIN) compared to proscillaridin A IC50 (24h) in 14 cancer cell lines. Correlation was evaluated by linear regression analysis; P-value is shown on the graph (n=3). C Representative pictures of transformed primary human fibroblasts before and after transduction with RASV12, MYC and RASV12/MYC were taken by light microscopy (400X magnification). D and E MYC expression was assessed by Western blotting in WT and MYC-transduced MOLT-4 cells (D) and REH cells (E). MYC expression was calculated as a ratio over ACTIN levels (*indicates P<0.05; One-way ANOVA; n = 3). IC50 values after 24h proscillaridin A treatment (ranging from 0.1 nM to 1 μM) in MOLT-4 cells (D) and REH cells (E) (n ≥ 3). F Time course experiment in NALM-6 cells treated with 5 nM for up to 96h. MYC expression was calculated as a ratio over ACTIN levels (*indicates P<0.05; One-way ANOVA; n = 3). (PDF 1640 kb) [file 13046_2019_1242_MOESM2_ESM.pdf]

**Figure S2**

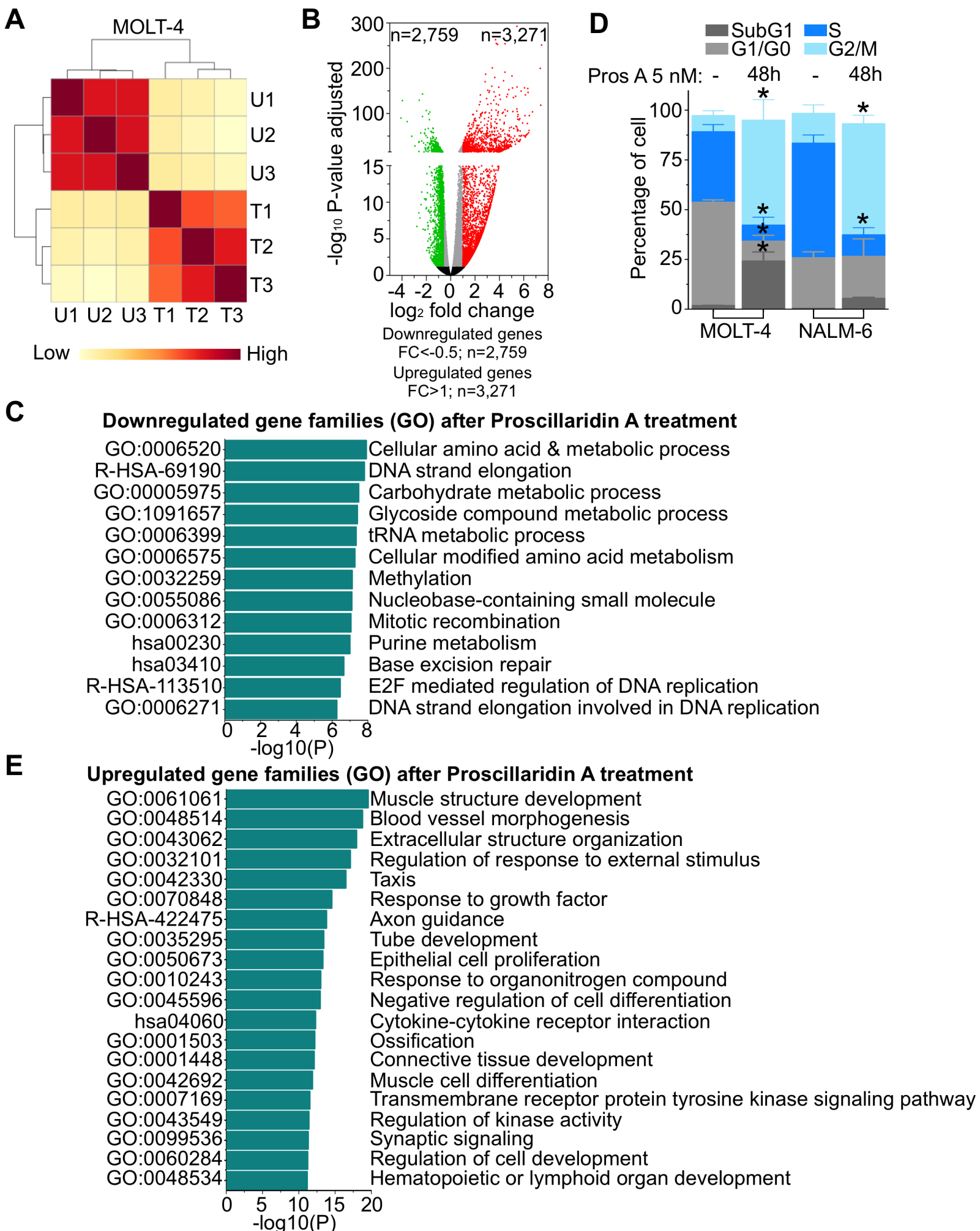

Supplement: Supplementary file 3 — Figure S2. Transcriptomic Analysis In MOLT-4 Cells Treated with Proscillaridin A (5 nM, 48h). A Heat map representing RPKM similarities between triplicates of untreated (U) and Proscillaridin A-treated (5 nM; 48h; T) MOLT-4 cells (n = 3). Red color corresponds to the highest similarity and yellow corresponds to the lowest similarity. B Proscillaridin A (5 nM, 48h) induced gene expression reprogramming of MOLT-4 cells. Volcano plots of gene expression changes in MOLT-4 cells in untreated versus treated samples. Black dots correspond to genes with P-value adjusted > 0.5. Grey dots correspond to genes with P-value adjusted < 0.5 but without significant fold change expression difference between untreated and treated cells (-0.5 < FC < 1). Downregulated genes with P-value adjusted < 0.5 and FC < -0.5 are shown in green. Upregulated genes with P-value adjusted < 0.5 and FC > 1 are shown in red. Numbers of downregulated and upregulated genes are shown on the graphs. C Metascape analysis of genes downregulated by proscillaridin A treatment (5 nM; 48h). D Cell cycle analysis after BrdU staining in MOLT-4 and NALM-6 cell lines exposed to proscillaridin A (5 nM, 48h). Cell fluorescence was measured by flow cytometry (* indicates P<0.05; Two-way ANOVA; n=3). E Metascape analysis of genes upregulated by proscillaridin A treatment (5 nM; 48h). (PDF 905 kb) [file 13046_2019_1242_MOESM3_ESM.pdf]

**Figure S3**

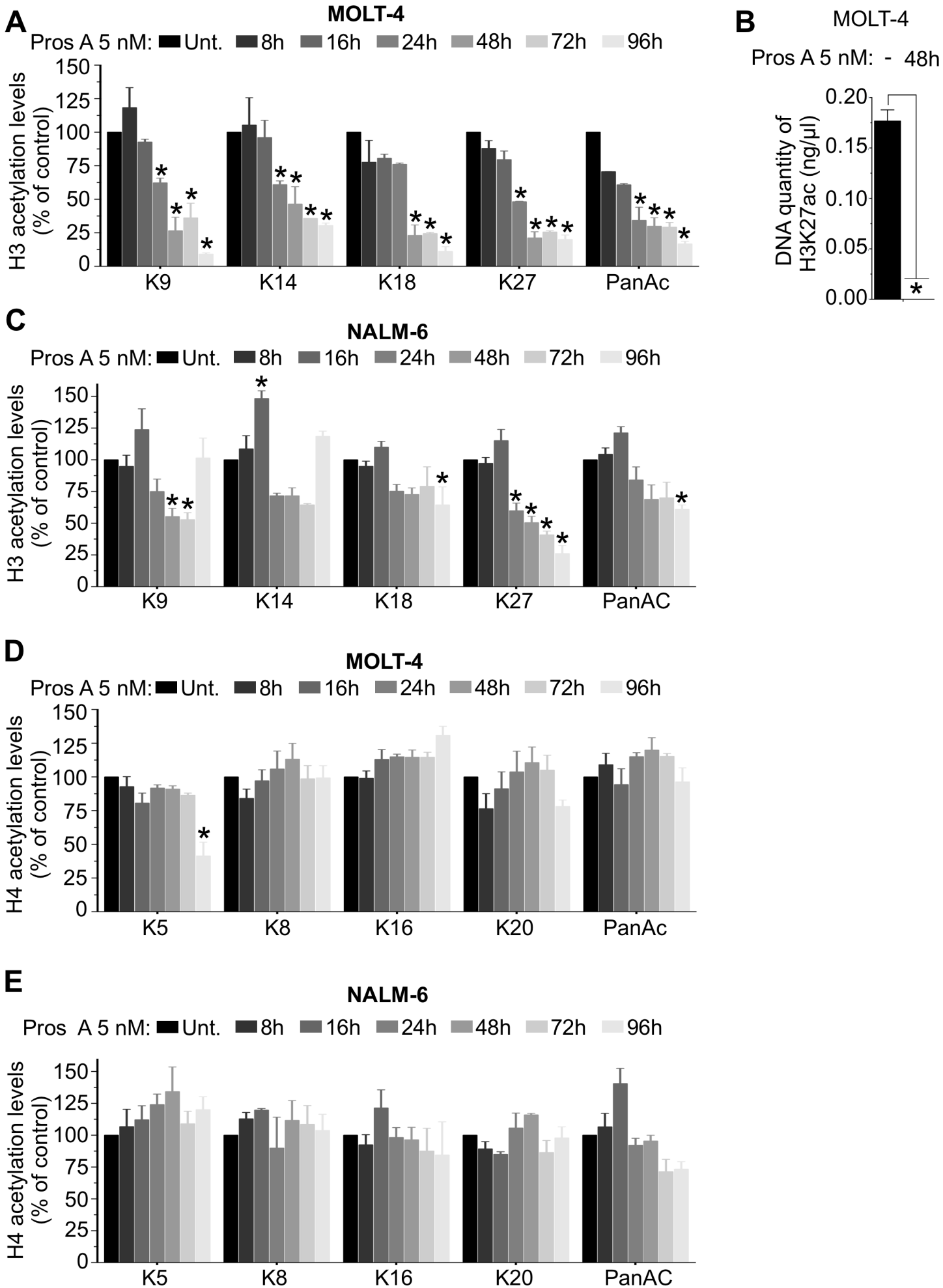

Supplement: Supplementary file 4 — Figure S3. Proscillaridin A Induced Histone 3 Acetylation Loss In MOLT-4 And NALM-6 Cells. A MOLT-4 cells were treated with proscillaridin A (5 nM) and histones were acid-extracted after 8, 16, 24, 48, 72 and 96 hours. H3 acetylation levels were quantified and expressed as a percentage of untreated cells (* indicates P<0.05; Two-way ANOVA; n = 3). B Ratio of chromatin immunoprecipitation (ChIP) of H3K27 acetylation in MOLT-4 cells before and after proscillaridin A treatment (5 nM; 48h) (*indicates P<0.001; paired t-test, n=3). C NALM-6 cells were treated with proscillaridin A (5 nM) and histones were acid-extracted after 8, 16, 24, 48, 72 and 96 hours. H3 acetylation levels were quantified and expressed as a percentage of untreated cells (* indicates P<0.05; Two-way ANOVA; n = 3). D MOLT-4 and E NALM-6 cells were treated with proscillaridin A (5 nM) and histones were acid-extracted after 8, 16, 24, 48, 72 and 96 hours. Histone 4 acetylation levels were assessed using antibodies against K5ac, K8ac, K16ac, K20ac, and total histone 4 acetylation. H4 was used as loading control. H4 acetylation levels were quantified and expressed as a percentage of untreated cells (* indicates P<0.05; Two-way ANOVA; n = 3). (PDF 567 kb) [file 13046_2019_1242_MOESM4_ESM.pdf]

**Figure S4**

**A**

**MOLT-4**

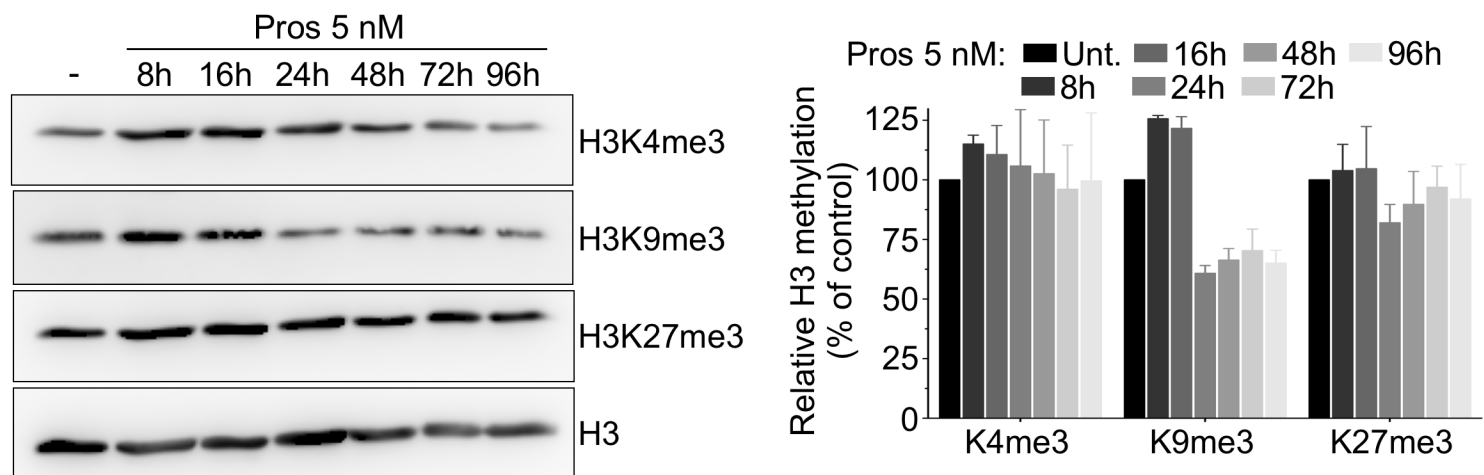

**B**

**NALM-6**

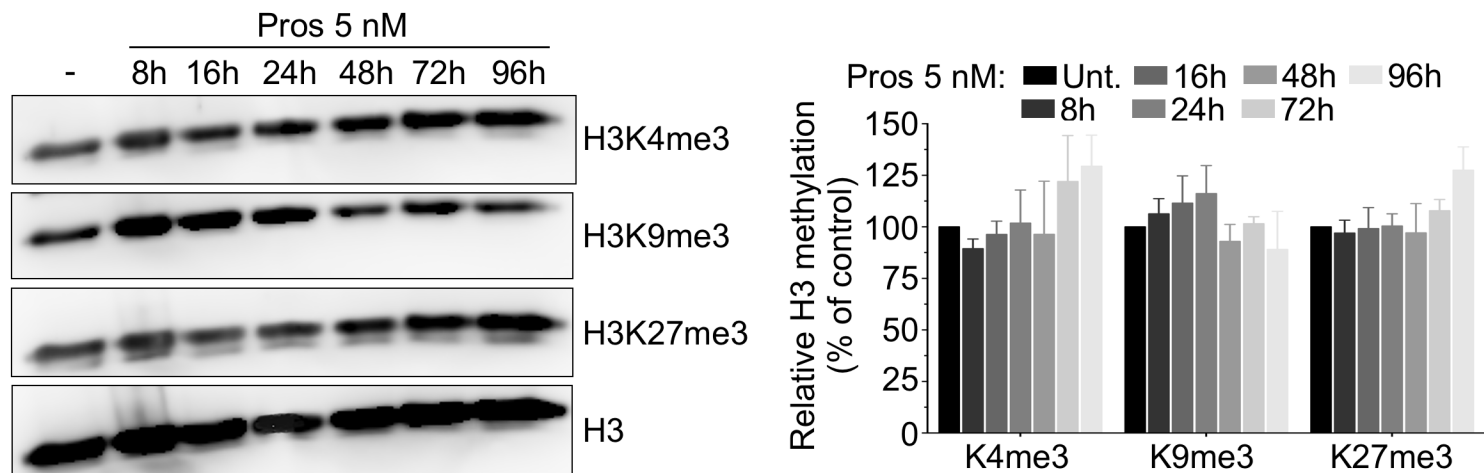

**C**

**MOLT-4 - DAPI**

Pros A 5 nM:

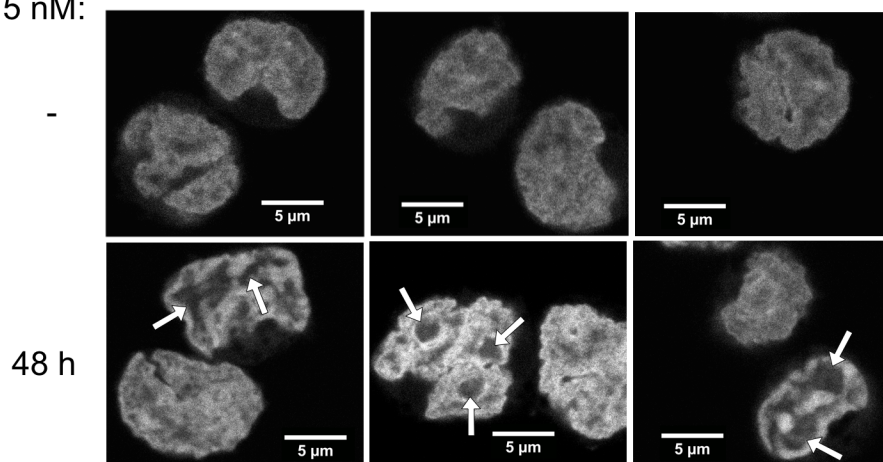

Supplement: Supplementary file 5 — Figure S4. Histone Methylation Is Not Significantly Altered After Proscillaridin A Treatment On Histone H3. MOLT-4 (A) and NALM-6 (B) cells were treated with proscillaridin A (5 nM) and histones were acid-extracted after 8, 16, 24, 48, 72 and 96 hours. Histone 3 methylation levels were assessed using antibodies against K4me3, K9me3, and K27me3. H3 was used as loading control. H3 methylation levels were quantified and expressed as a percentage of untreated cells (Two-way ANOVA; n = 3). C Confocal microscopy (60X) of MOLT-4 cells stained with DAPI revealed heterochromatin modulation after proscillaridin A treatment (5 nM; 48h). White arrows indicate loss of heterochromatin regions. (PDF 1592 kb) [file 13046_2019_1242_MOESM5_ESM.pdf]

**Figure S8****A**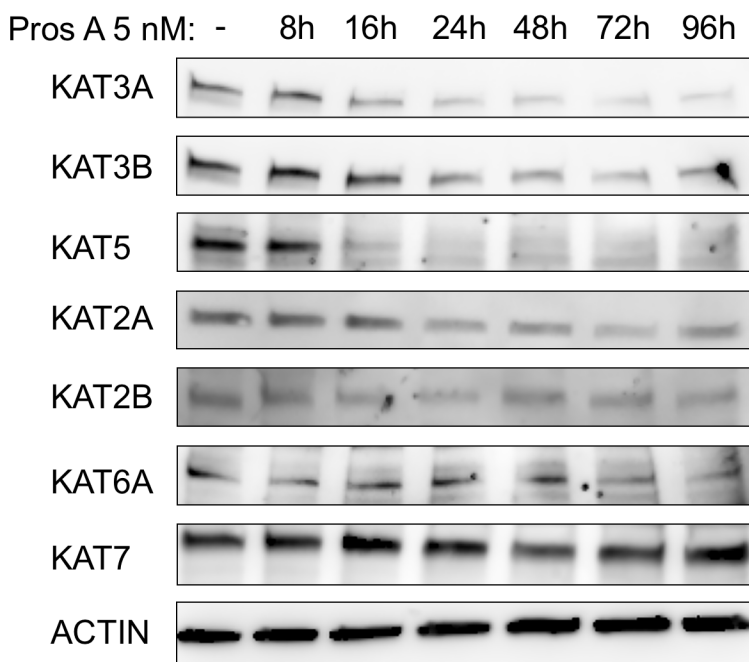**B**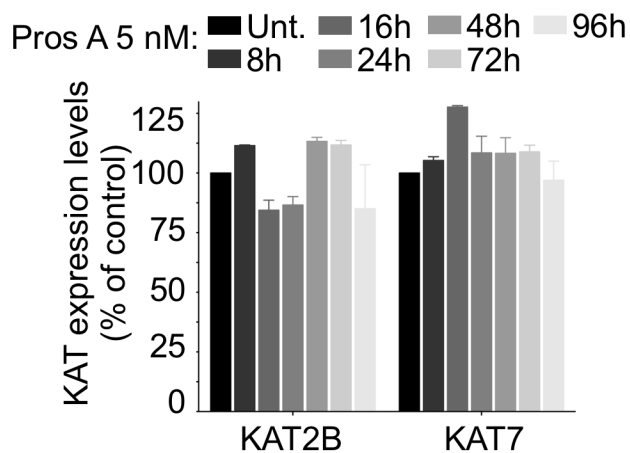**C**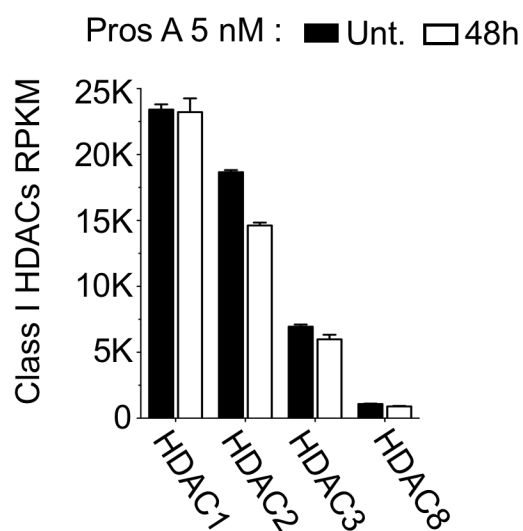**D**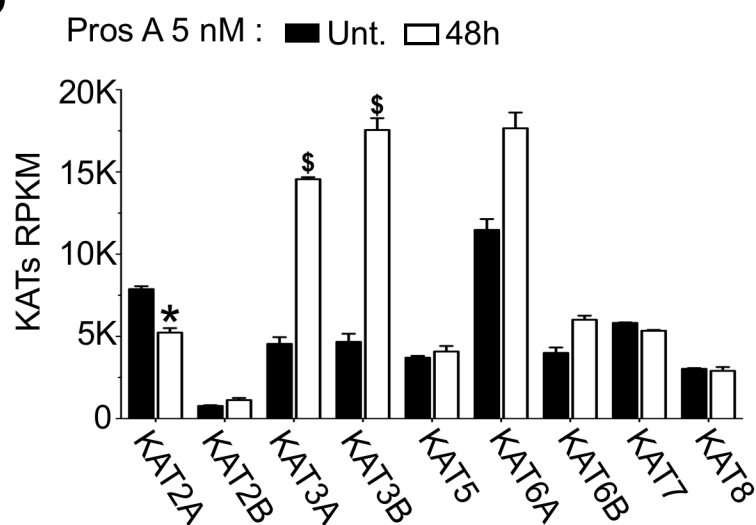**E**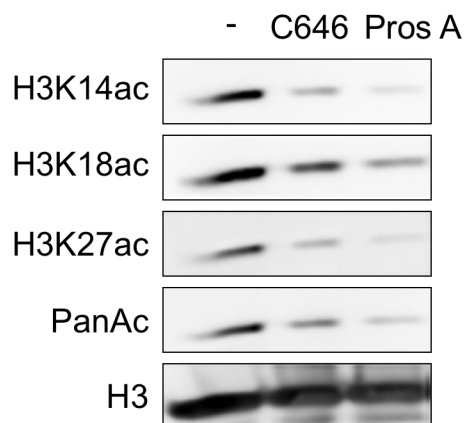**F**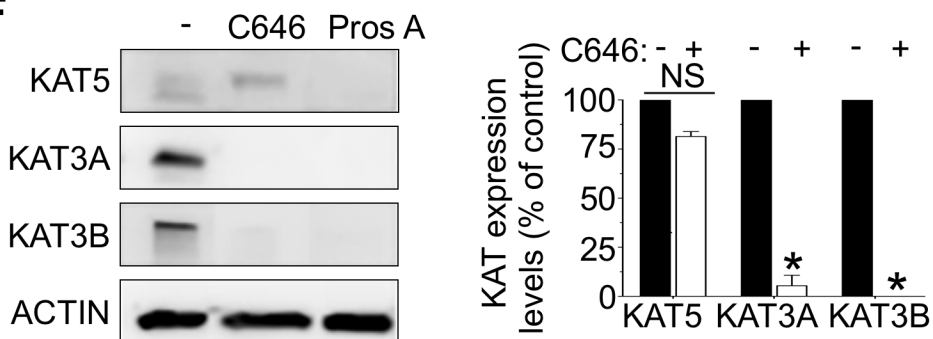

Supplement: Supplementary file 9 — Figure S8. MYC Inhibition Induced By Proscillaridin A Is Regulated By KAT Activities. A MOLT-4 cells were treated with proscillaridin A (5 nM) and KAT3A, KAT3B, KAT5, KAT2A, KAT2B, KAT6A and KAT7 expression levels were assessed by western blotting. ACTIN was used as loading control. B KAT2B and KAT7 expression levels were quantified and expressed as percentage of untreated cells (n=3). C and D Class I HDAC (C) and KAT (D) expression transcripts (RPKM) expression after proscillaridin A treatment (5 nM; 48h) in RNA-sequencing data set (*indicates Log2 FC<-0.5 and $ indicates Log2 FC > 1). E MOLT-4 cells were treated with KAT3B/A inhibitor C646 (10 μM) and with proscillaridin A (5 nM) and histones were acid-extracted after 48 hours. Histone 3 acetylation levels were assessed using antibodies against K14ac, K18ac, K27ac, and pan histone 3 acetylation. H3 was used as loading control. F Left panel, MOLT-4 cells were treated with KAT3B/A inhibitor C646 (10 μM) and with Proscillaridin A (5 nM) and KAT5, KAT3A and KAT3B expression levels were assessed by western blotting. ACTIN was used as loading control. Right panel, KAT5, KAT3A and KAT3B levels were quantified and expressed as a percentage of untreated cells (* indicates P<0.05; Two-way ANOVA; n = 3). (PDF 1077 kb) [file 13046_2019_1242_MOESM9_ESM.pdf]

**Figure S9**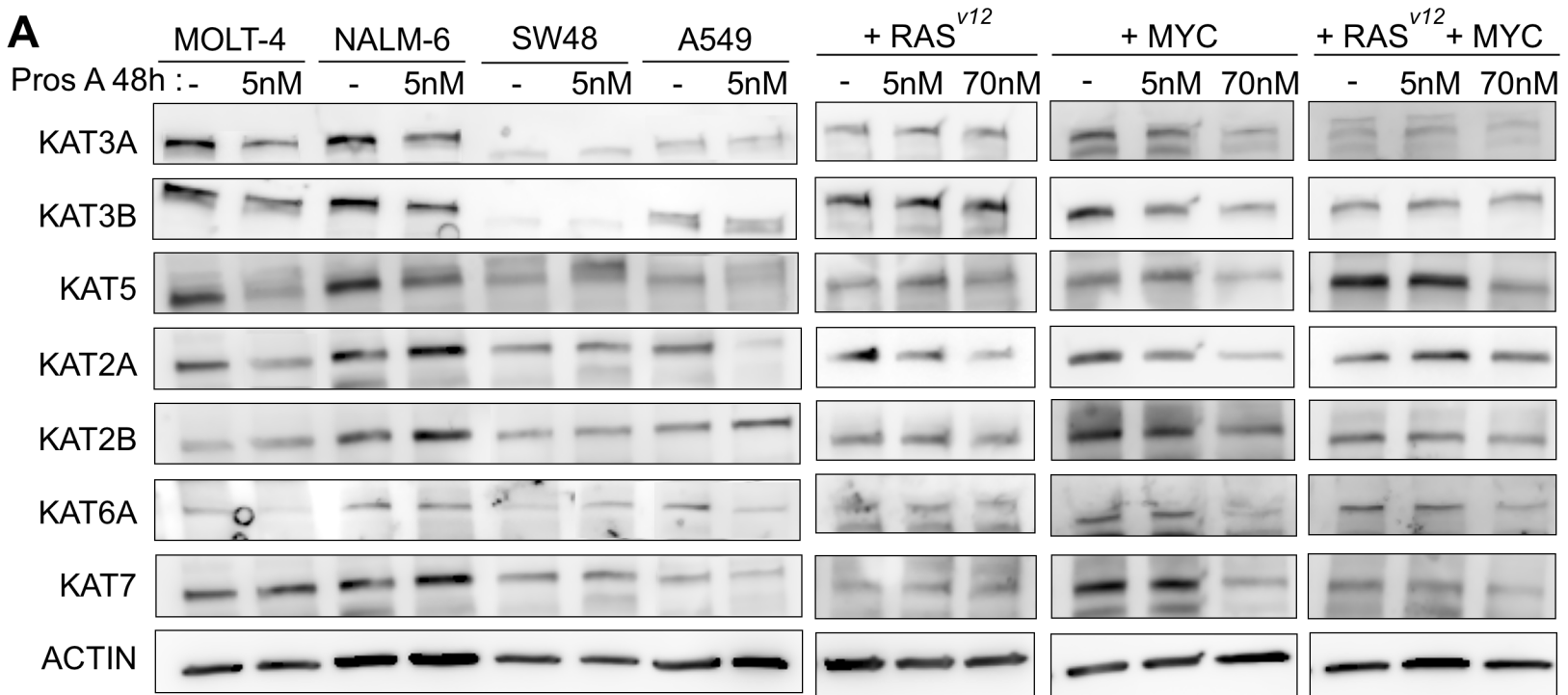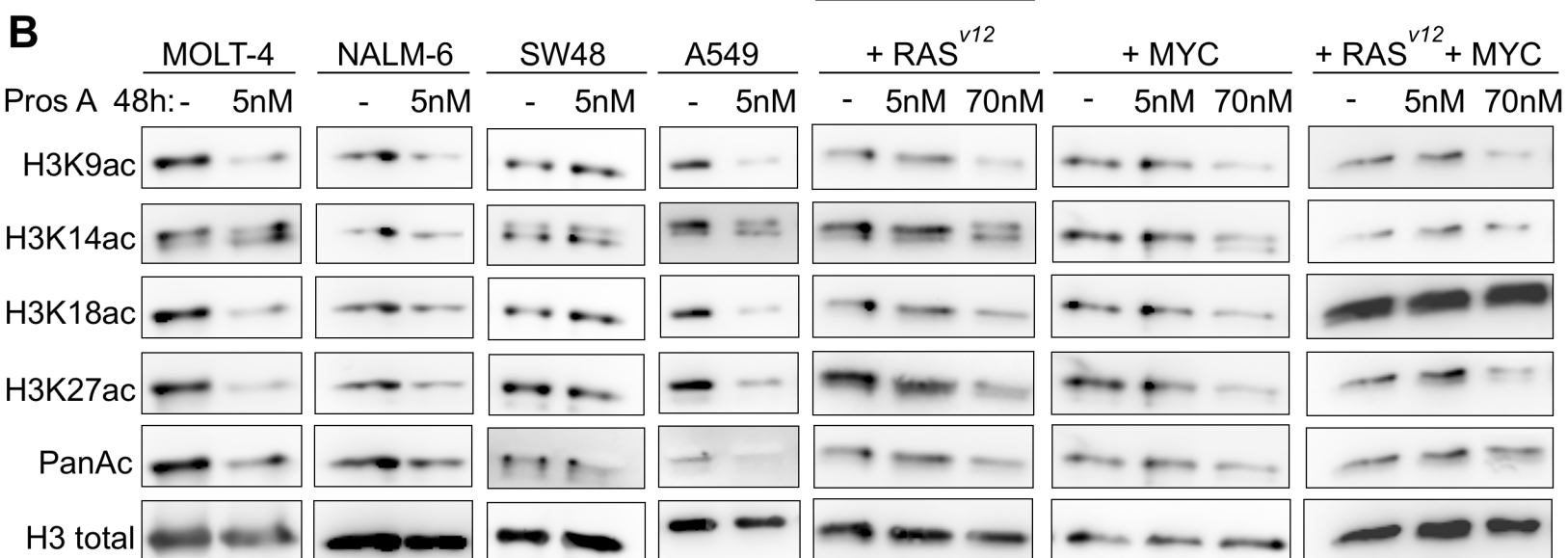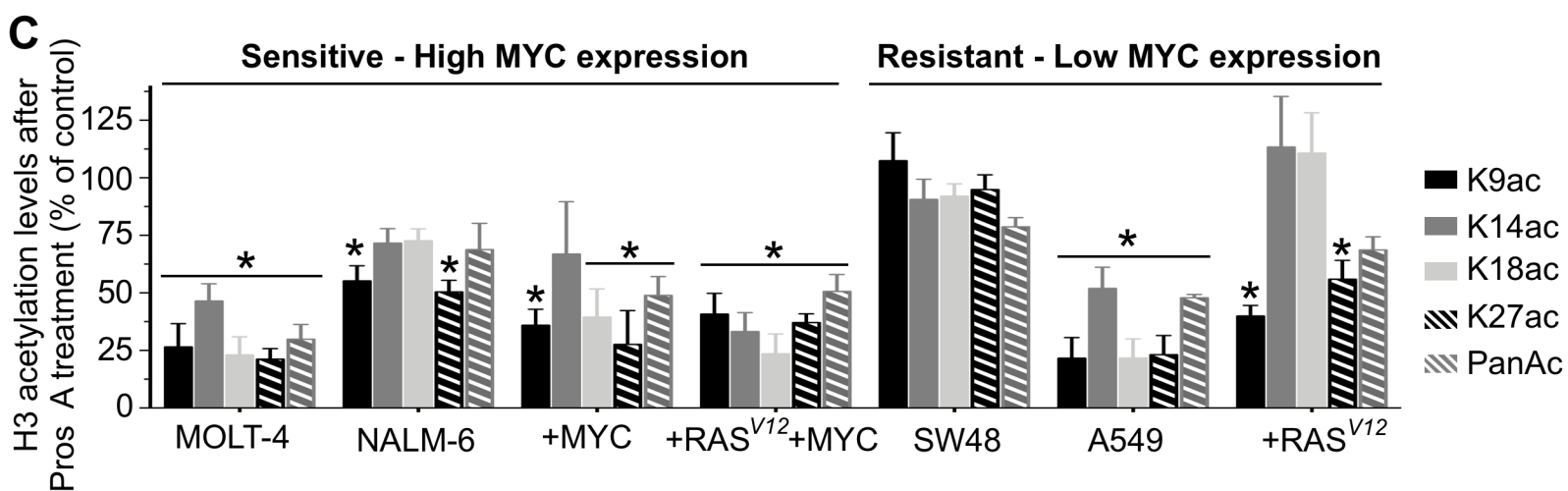

Supplement: Supplementary file 10 — Figure S9. Proscillaridin A Induces KAT Downregulation Specifically In High MYC Expressing Cells. A-C MOLT-4, NALM-6, SW48 and A549 cell lines were treated with proscillaridin A (5 nM, 48h) and fibroblasts transduced with RASV12, MYC and RASV12/MYC were treated with proscillaridin A (5 nM or 70 nM, 48h). A KAT3A (CBP), KAT3B (P300), KAT5 (TIP60), KAT2A (GCN5), KAT2B (PCAF), KAT6A (MOZ) and KAT7 (HBO1) expression levels were assessed by western blotting. ACTIN was used as loading control. B Histone 3 acetylation levels were assessed by using antibodies against K9ac, K14ac, K18ac, K27ac, and pan histone 3 acetylation. H3 total was used as loading control. C Histone 3 acetylation levels were quantified and expressed as percentage of control (* indicates P<0.05; One-way ANOVA; n=3). (PDF 2285 kb) [file 13046_2019_1242_MOESM10_ESM.pdf]
