# Supplementary material for: Heart failure drug proscillaridin A targets MYC overexpressing leukemia through global loss of lysine acetylation
Source: J Exp Clin Cancer Res. 2019 Jun 13;38:251. doi: 10.1186/s13046-019-1242-8 (PMC6563382; doi:10.1186/s13046-019-1242-8)
Supplement: Supplementary file 6 — Figure S5. H3K27 Acetylation DNA Occupancy Is Lost After Proscillaridin A Treatment In MOLT-4 Cells. Metascape analysis of A downregulated genes and B upregulated genes after proscillaridin A treatment (5 nM; 48h) marked by H3K27ac in their promoter regions (-500 bp / +500 bp). (PDF 657 kb) [file 13046_2019_1242_MOESM6_ESM.pdf]

Figure S5

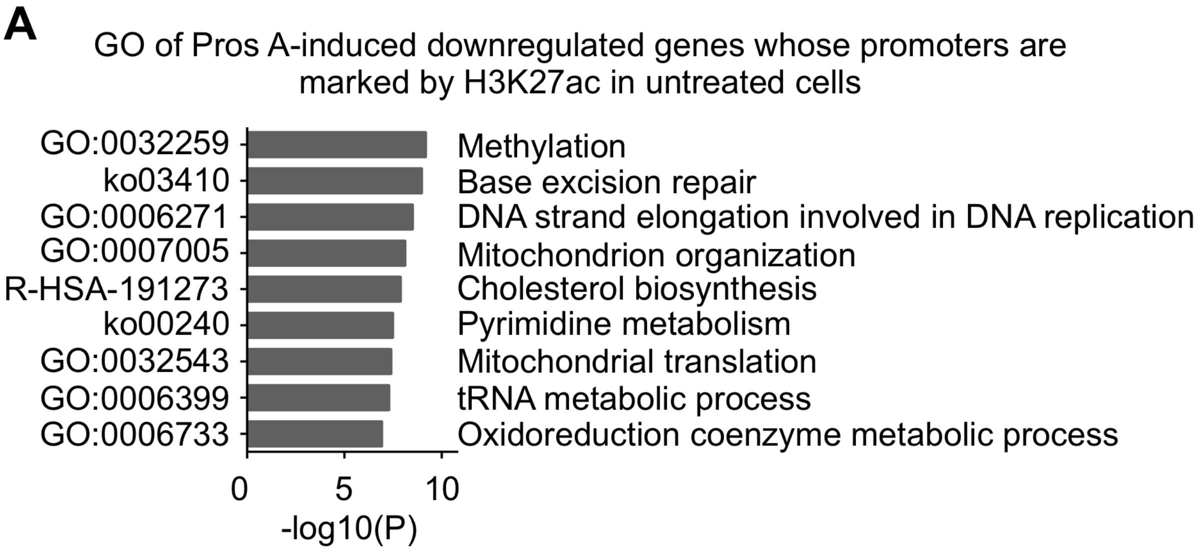
A
GO of Pros A-induced downregulated genes whose promoters are marked by H3K27ac in untreated cells
GO:0032259
ko03410
GO:0006271
GO:0007005
R-HSA-191273
ko00240
GO:0032543
GO:0006399
GO:0006733
Methylation
Base excision repair
DNA strand elongation involved in DNA replication
Mitochondrion organization
Cholesterol biosynthesis
Pyrimidine metabolism
Mitochondrial translation
tRNA metabolic process
Oxidoreduction coenzyme metabolic process
0
5
10
-log10(P)

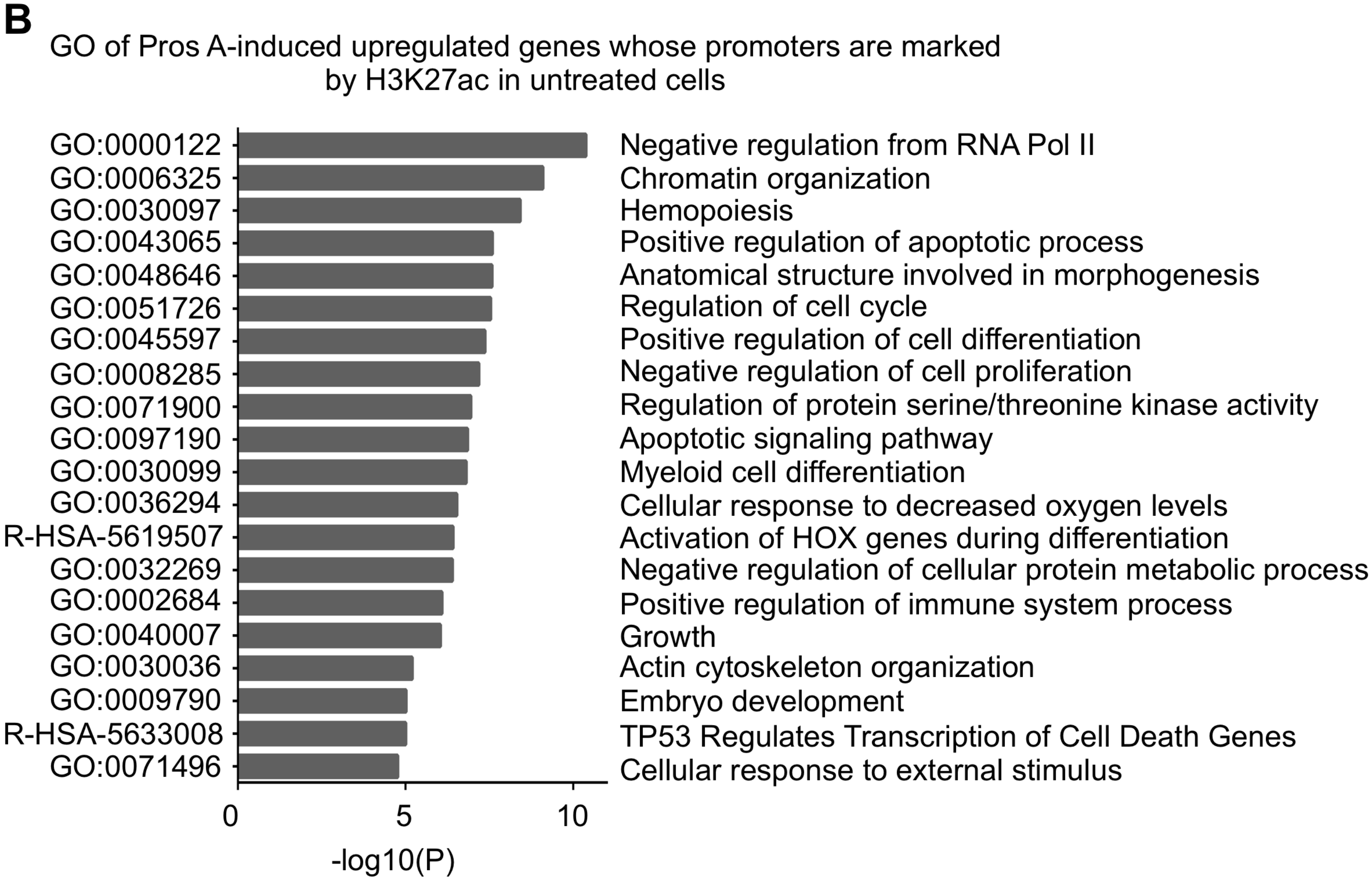
B
GO of Pros A-induced upregulated genes whose promoters are marked by H3K27ac in untreated cells
GO:0000122
GO:0006325
GO:0030097
GO:0043065
GO:0048646
GO:0051726
GO:0045597
GO:0008285
GO:0071900
GO:0097190
GO:0030099
GO:0036294
R-HSA-5619507
GO:0032269
GO:0002684
GO:0040007
GO:0030036
GO:0009790
R-HSA-5633008
GO:0071496
Negative regulation from RNA Pol II
Chromatin organization
Hemopoiesis
Positive regulation of apoptotic process
Anatomical structure involved in morphogenesis
Regulation of cell cycle
Positive regulation of cell differentiation
Negative regulation of cell proliferation
Regulation of protein serine/threonine kinase activity
Apoptotic signaling pathway
Myeloid cell differentiation
Cellular response to decreased oxygen levels
Activation of HOX genes during differentiation
Negative regulation of cellular protein metabolic process
Positive regulation of immune system process
Growth
Actin cytoskeleton organization
Embryo development
TP53 Regulates Transcription of Cell Death Genes
Cellular response to external stimulus
0
5
10
-log10(P)
