# Supplementary material for: Heart failure drug proscillaridin A targets MYC overexpressing leukemia through global loss of lysine acetylation
Source: J Exp Clin Cancer Res. 2019 Jun 13;38:251. doi: 10.1186/s13046-019-1242-8 (PMC6563382; doi:10.1186/s13046-019-1242-8)

Figure S6

A

Map of co-expression pathways of MYC target genes downregulated by Pros A (5 nM; 48h) treatment and marked by H3K27ac in untreated cells

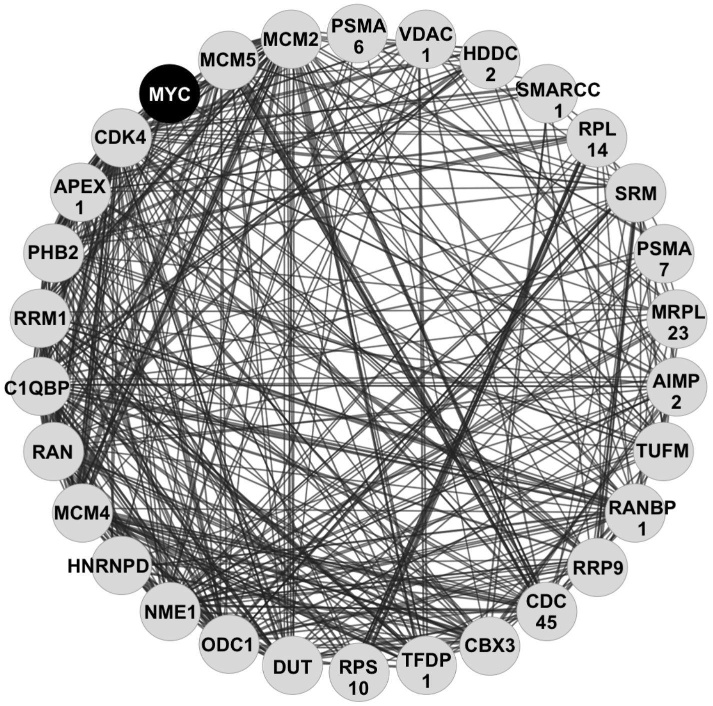

B

Map of physical interactions of MYC target genes downregulated by Pros A (5nM; 48h) treatment and marked by H3K27ac in untreated cells

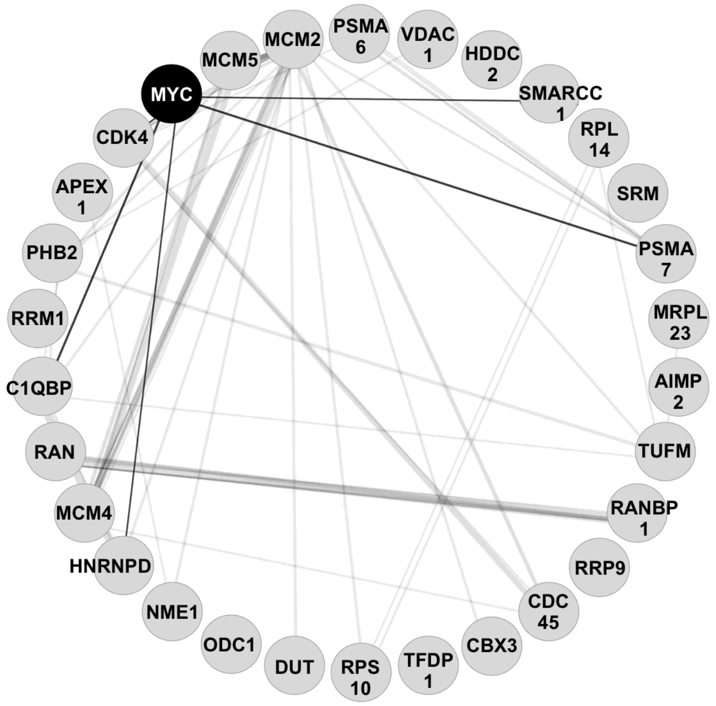

Supplement: Supplementary file 7 — Figure S6. Proscillaridin A Treatment Downregulated MYC Target Genes That Are Marked By H3K27ac In Promoter Regions. Map of A co-expression pathways and B protein-protein physical interactions of MYC target genes marked by H3K27ac in untreated MOLT-4 cells. (PDF 1418 kb) [file 13046_2019_1242_MOESM7_ESM.pdf]
