# Supplementary material for: Heart failure drug proscillaridin A targets MYC overexpressing leukemia through global loss of lysine acetylation
Source: J Exp Clin Cancer Res. 2019 Jun 13;38:251. doi: 10.1186/s13046-019-1242-8 (PMC6563382; doi:10.1186/s13046-019-1242-8)

Figure S7

A

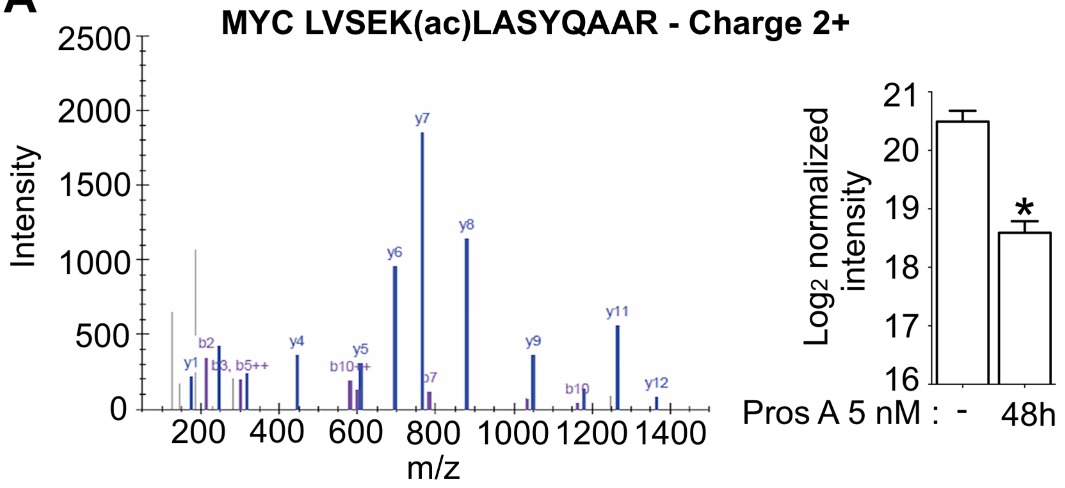

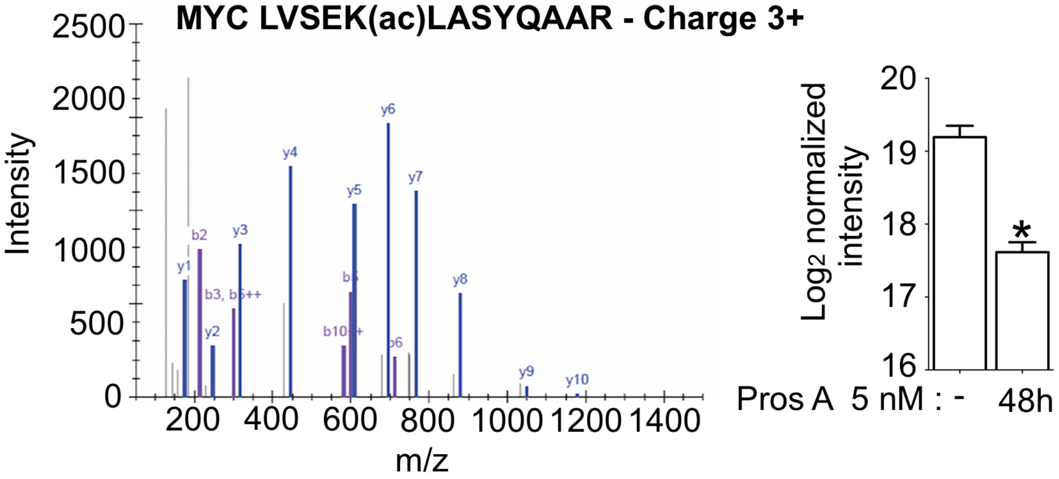

B

Map of co-expression pathways of MYC targets losing acetylation after Pros treatment

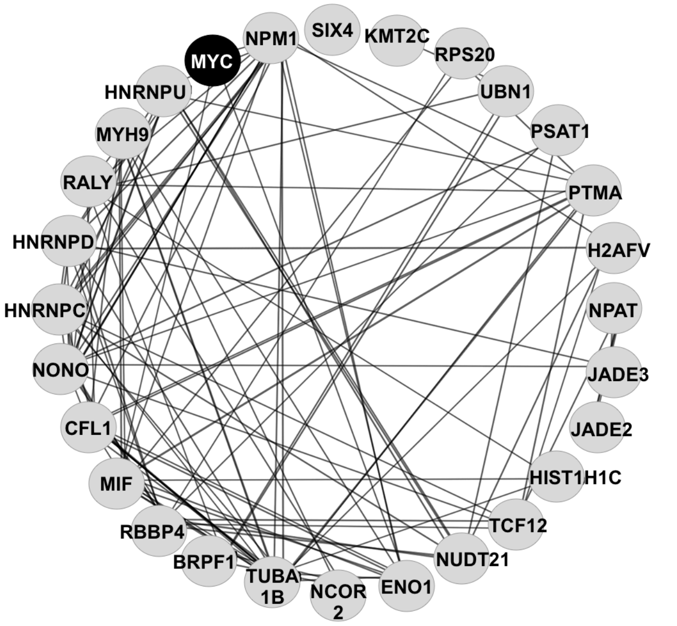

Supplement: Supplementary file 8 — Figure S7. Loss of Acetylation In MYC Protein And MYC After Proscillaridin A Treatment In High MYC Expressing Cells. A Mass spectrometry analysis on 2 MYC peptides (LVSEK(ac)LASYQAAR) after proscillaridin A treatment (5nM; 48h) in MOLT-4. Log2 normalized intensity is shown (* indicates P<0.001; paired t-test; n=4). B Map of co-expression pathways of the 28 proteins that lost acetylation after proscillaridin A treatment (5 nM; 48h) in MOLT-4 cells. (PDF 890 kb) [file 13046_2019_1242_MOESM8_ESM.pdf]
